# Cognitive and Behavioral Outcomes of Snoring Among Adolescents

**DOI:** 10.1001/jamanetworkopen.2024.44057

**Published:** 2024-11-08

**Authors:** Amal Isaiah, Sophia Uddin, Thomas Ernst, Christine Cloak, Dongdong Li, Linda Chang

**Affiliations:** 1 Department of Otorhinolaryngology–Head and Neck Surgery, University of Maryland School of Medicine, Baltimore; 2 Department of Pediatrics, University of Maryland School of Medicine, Baltimore; 3 Department of Diagnostic Radiology and Nuclear Medicine, University of Maryland School of Medicine, Baltimore; 4 University of Maryland Institute for Health Computing, Bethesda; 5 Department of Population Medicine, Harvard Medical School, Harvard Pilgrim Health Care Institute, Boston, Massachusetts; 6 Department of Biostatistics, Harvard T.H. Chan School of Public Health, Boston, Massachusetts; 7 Department of Neurology, University of Maryland School of Medicine, Baltimore; 8 Department of Neurology, Johns Hopkins University School of Medicine, Baltimore, Maryland

## Abstract

**Importance:**

Snoring is central to sleep-disordered breathing (SDB), which arises from nocturnal upper airway resistance. Habitual snoring is associated with cognitive and behavioral problems in young children, but less is known about these associations in adolescents.

**Objective:**

To assess the longitudinal associations of snoring with cognition and problem behaviors among adolescents.

**Design, Setting, and Participants:**

This cohort study was a secondary analysis of the Adolescent Brain Cognitive Development (ABCD) Study dataset (release 5.0), which enrolled 11 875 children and a parent or caregiver from June 1, 2016, to October 15, 2018, at 21 participating US research institutions and followed-up for 5 years. The analysis was performed between December 2023 and April 2024.

**Exposures:**

Parent-reported snoring categorized as none, nonhabitual (<3 nights/week), and habitual (≥3 times/week).

**Main Outcomes and Measures:**

Cognition was assessed using 5 measures from the National Institutes of Health Toolbox (NIH-TB), and caregiver-reported problem behaviors were assessed using the Child Behavior Checklist (CBCL), including Total Problems, Internalizing Problems, and Externalizing Problems. Longitudinal associations of snoring with these assessment measures were analyzed using linear mixed-effects models stratified by obesity. Model fits were assessed after including the fixed-effects of age, sex at birth, race, family income, follow-up time, visit type, and the random-effects of site and identification number.

**Results:**

The study included data from 11 862 children at year 1 (mean age, 119.0 months [95% CI, 118.8 to 119.1 months]; 6164 male [52.2%]) and 11 198, 10 870, 10 064, and 4668 children at years 2 to 5, respectively. The proportion of habitual snorers decreased from 811 participants (6.8%) in year 1 to 150 participants (3.2%) in year 5. Snoring was not associated with any of the NIH-TB scores. A statistically significant association of snoring was identified with all CBCL scales. The largest-magnitude association was of snoring with the CBCL Total Problems scale among adolescents with obesity (β = 3.18; 95% CI, 2.59-3.77).

**Conclusions and Relevance:**

In this cohort study analyzing associations of snoring with cognitive test scores and problem behaviors based on the ABCD dataset, the prevalence of snoring declined over time; snoring in adolescents was associated with problem behaviors but not cognitive deficits. These findings may aid shared decision-making regarding treatment options such as adenotonsillectomy for adolescents with SDB.

## Key Points

**Question:**

Is parent-reported snoring frequency associated with cognitive and behavioral outcomes in adolescents?

**Findings:**

In this cohort study of 11 862 adolescents, frequent snoring was associated with greater problem behaviors but not with lower cognition.

**Meaning:**

These findings suggest that clinicians should incorporate the differential associations of snoring with cognitive and behavioral outcomes in shared decision-making concerning the management of adolescents with sleep-disordered breathing symptoms.

## Teaser

This longitudinal cohort study analyzes associations of parent-reported frequency of snoring with cognitive and behavioral outcomes in adolescents.

## Introduction

Snoring results from impaired airflow through the upper airway during sleep and is the most consistently observed symptom of obstructive sleep-disordered breathing (SDB) in children.^1^ The most common factors associated with increased risk for snoring in children are adenotonsillar hypertrophy and obesity. While snoring in otherwise healthy children was historically considered benign, nearly all clinical societies advocate for the early evaluation and management of snoring. The recommended management of snoring varies by location. Clinical societies in the US uniformly recommend a proactive approach toward habitual snoring, defined as snoring 3 or more nights per week. For example, recognizing that habitual snoring is not benign, the American Academy of Pediatrics recommends polysomnography or referral to an otolaryngologist or sleep medicine physician for further management.^2^ The American Academy of Sleep Medicine also emphasizes this position.^3^ However, the American Academy of Otolaryngology reserves polysomnography only for children at high risk of adenotonsillectomy-related complications or those with less certain diagnoses, leaving room for a preference-sensitive approach to adenotonsillectomy as a potential management of habitual snoring and SDB.^4^ The European Respiratory Society also recommends further evaluation of snoring using polysomnography.^5^ Other recommendations from China,^6^ Australia,^7^ and India,^8^ and combined data from the Asia-Pacific^9^ also emphasize the need for further evaluation and management of habitual snoring.

A large body of literature has observed negative associations of SDB with neurobehavioral outcomes, thus supporting cognition and behavior as key outcomes of the only randomized clinical trials in the domain, the Pediatric Adenotonsillectomy for Snoring^10^ and the Childhood Adenotonsillectomy Trial.^11^ Although polysomnography is central to the objective stratification of SDB, habitual snoring is now recognized as an essential marker of SDB morbidity in children.^9^ The importance of this association is that due to the shortage of polysomnography, parent-reported symptoms such as snoring frequency can be used to prioritize interventions such as adenotonsillectomy.^7,11^ More importantly, snoring was also identified as a key symptom associated with cognitive and behavioral deficits in snoring children, independent of polysomnographic findings.^12^ Regardless of its definition, SDB is associated with poor academic performance, specifically language, arts, math, science, and learning problems.^13^ Finally, a recent, comprehensive, and high-quality meta-analysis of 63 studies demonstrated multiple deficits across cognitive domains in children with SDB, especially within the full-scale intelligence quotient and the subdomains of problem-solving, working memory, processing speed, and language.^14^ These findings reinforce that when snoring is used as a population-based screening measure, it traverses the spectrum of SDB from primary snoring to the most severe SDB, including obstructive sleep apnea.

Most SDB-related morbidity and treatment studies have focused on preschool- or young elementary school–aged children. While some large population-based studies and other longitudinal studies contained a small number of adolescents, only a few have examined the natural history of SDB in adolescents.^15^ Furthermore, adolescence is a period of rapid changes in craniofacial anatomy and brain development. However, it remains unclear whether these adolescents have similar neurobehavioral impacts from SDB as they would when their brain development is at a substantially younger critical period.^16^ The lack of SDB morbidity data from adolescents makes it challenging to determine whether parent-reported symptoms of SDB remain relevant for initiating management options such as adenotonsillectomy or other nonsurgical options. Given this context, the Adolescent Brain Cognitive Development (ABCD) Study^7^ provides an excellent opportunity to probe the associations of SDB with adolescent brain development. Parent-reported symptoms of SDB projected children’s behavior, which was associated with thinner prefrontal cortices, brain regions responsible for attention and executive function.^18,19^

Given these earlier findings, we sought to use the subsequent waves of the ABCD dataset to dissect further the association of snoring with cognition and behavior in adolescents using robust longitudinal models. We expect our findings to fill the void in longitudinal data from adolescents with SDB. Theoretically, in children who continue to snore as they age, the cumulative negative effects of snoring could worsen cognition and behavior over time. Therefore, we hypothesized that adolescents who continued to snore throughout our study would show slower cognitive and behavioral development or deterioration of these measures over time.

## Methods

### Study Design and Participants

This cohort study was determined exempt by the University of Maryland, Baltimore institutional review board and followed the Strengthening the Reporting of Observational Studies in Epidemiology (STROBE) reporting guideline. This study is based on release 5.0 of the ABCD dataset obtained from the National Institute of Mental Health Data Archive. The ABCD Study is a large prospective cohort study of 11 875 children enrolled between June 1, 2016, to October 15, 2018, at 21 participating US research institutions.^17^ One or both parents or a guardian provided written informed consent; assent was obtained from children capable of expressing their willingness to participate. The children in the current study were enrolled and evaluated at 9 to 10 years of age and reassessed annually between 2016 and 2021. The sample size and the projected attrition over time allow for detection of small to medium effect sizes for most exposures involving child development from preadolescence to adulthood.^20^

### Exposure, Outcomes, and Covariates

Snoring was derived from the Sleep Disturbance Scale for Children, a 27-item, validated questionnaire for sleep problems in children.^21^ The survey includes a 5-point qualitative assessment of snoring with the following categories: no snoring (1), occasional snoring (2; 1-2 nights per month), sometimes snoring (3; 1-2 nights per week), often snoring (4; >2 nights per week but not daily), and daily snoring (5). In line with published literature, we recategorized the response to the question into the following: no snoring, nonhabitual snoring (1-2 nights per week), and habitual snoring (≥3 nights per week).^9,12,22,23^

Cognition was assessed using the National Institutes of Health Toolbox (NIH-TB) for Assessment of Neurological and Behavioral Function Cognitive Battery, which has robust psychometric properties. We included the 5 tests that could be conducted remotely during the COVID-19 pandemic and, therefore, had longitudinal data. All tests were administered on a tablet and evaluated executive function, episodic visual memory, immediate verbal recall, language, processing speed, working memory, and cognitive control or attention.^24^ Specifically, the Picture Vocabulary and Oral Reading Recognition tests measure language skills. The Flanker Inhibitory Control and Attention Test evaluates the ability to ignore distractors. The Pattern Comparison Processing Speed Test assesses processing speed by asking participants to compare 2 pictures. In the Picture Sequence Memory Test, participants are shown images in a specific sequence and a corresponding narration. They are then asked to recall and recreate the original order of the pictures. The Crystallized Cognition composite score uses Picture Vocabulary and Oral Reading Recognition scores. The NIH-TB was administered to the participants in years 1, 3, and 5. Uncorrected scores were used for analysis.

Problem behaviors were evaluated through parental responses to the Child Behavior Checklist (CBCL), an established tool for assessing childhood behavior, including emotional, social, and behavioral domains.^25^ The specific syndrome scales measured were Anxious or Depressed, Somatic Complaints, Social Problems, Thought Problems, Attention Problems, Rule-Breaking Behavior, and Aggressive Behavior. These items were aggregated into Internalizing Problems, Externalizing Problems, and Total Problems scales. To standardize these raw scores, they were converted to T scores based on sex and age norms derived from population studies, with higher T scores indicating more severe problem behaviors. The CBCL was administered yearly.

Other study variables included age in months, sex assigned at birth, self-selected race (White, Black, and other [defined as any race not otherwise specified]), time point (years 1-5, representing baseline to 4-year follow-up), participant identification (ID) number, and site. Additionally, the body mass index (BMI) percentile was calculated using the formula of weight in kilograms divided by height in meters squared on age- and sex-specific growth charts provided by the US Centers for Disease Control and Prevention.^26^ A BMI for age greater than the 95th percentile was considered obese.^27^ Total household pretax income was included as an ordinal variable in 10 categories ranging from less than $5000 to greater than $200 000. Because income and educational status are highly correlated in the ABCD dataset, only income was included to preserve parsimony by preventing variable inflation. Given the COVID-19 pandemic–related changes in testing formats, the testing format (in-person, remote, and hybrid) was also included.

### Statistical Analysis

Distribution of the outcomes stratified by snoring status across time points was visualized by boxplots. The time-varying associations of snoring with cognitive and behavioral outcomes were assessed using linear mixed-effects regression models implemented using R version 4.3,(R Project for Statistical Computing).^28^ The linear mixed-effects model had snoring as the main exposure and was adjusted for age, sex assigned at birth, race, total pretax family income, and testing format. Random-effects included participant ID nested within site ID. Models were further assessed separately for children with a BMI less than the 95th percentile (nonobese) and at the 95th percentile or greater (obese) due to the known association of snoring with obesity.^29^ The interaction between time points and snoring was added to the model to evaluate the association of snoring with the outcomes over time. Mixed-effects models incorporated all data from study participants at all sites with at least 1 measurement across time points and account for missing data using a maximum-likelihood-based approach.^30,31^ A 2-sided *P* value of .05 was considered significant. Analysis was conducted from December 2023 to April 2024.

## Results

The study included 11 862 children at baseline (year 1; mean age, 119.0 months [95% CI, 118.8-119.1 months]; 6164 male [52.2%]; 1958 Black [16.6%]; 8699 White [73.6%]; 1163 other race [16.6%]), 11 198 in year 2, 10 870 in year 3, and 10 064 in year 4. Only one-half of the dataset was available in year 5, with 4688 participants. The demographic characteristics of the sample are shown in the Table. eFigure 1 and eFigure 2 in Supplement 1 demonstrate the extent of missingness in the ABCD dataset used in the current study.

**Table.** Demographic Characteristics of the Study Sample Over 5 Years

Variable	Participants, No. (%) (N = 11 875)				
	Year 1 (n = 11 862)	Year 2 (11,198)	Year 3 (n = 10 870)	Year 4 (n = 10 064)	Year 5 (n = 4668)
Age, mean (95% CI), mo	119.0 (118.8-119.1)	131.1 (130.9-131.2)	144.3 (144.1-144.4)	154.9 (154.8-155.1)	169.0 (168.7-169.2)
Sex^a^					
Male	6164 (52.2)	5836 (52.3)	5701 (52.5)	5303 (52.7)	2454 (52.5)
Female	5653 (47.8)	5323 (47.7)	5164 (47.5)	4768 (47.34)	2224 (47.54)
Race^b^					
White	8699 (73.6)	8338 (74.7)	8117 (74.7)	7659 (76.0)	3690 (78.9)
Black	1958 (16.6)	1755 (15.7)	1709 (15.7)	1467 (14.6)	553 (11.8)
Other	1163 (9.8)	1069 (9.6)	1042 (9.6)	947 (9.4)	436 (9.3)
BMI, percentile (95% CI)^c^	61.1 (60.5-61.6)	62.4 (61.8-62.9)	63.2 (62.6-63.9)	65.3 (64.0-65.3)	66.4 (65.4-67.4)
Yearly income, $					
<5000	410 (3.8)	361 (3.6)	338 (3.4)	227 (2.5)	114 (2.7)
5000-12 000	415 (3.9)	363 (3.6)	352 (3.6)	303 (3.3)	127 (3.0)
12 000-16 000	269 (2.5)	241 (2.4)	225 (2.3)	203 (2.2)	94 (2.2)
16 000-25 000	516 (4.8)	465 (4.6)	452 (4.6)	403 (4.4)	174 (4.1)
25 000-35 000	646 (6.0)	591 (5.8)	587 (5.9)	520 (5.7)	254 (5.9)
35 000-50 000	915 (8.5)	855 (8.4)	836 (8.4)	776 (8.5)	368 (8.9)
50 000-75 000	1483 (13.8)	1404 (13.8)	1361 (13.7)	1285 (14.0)	602 (14.0)
75 000-100 000	1551 (14.4)	1499 (14.8)	1479 (14.9)	1391 (15.2)	665 (15.5)
100 000-200 000	3279 (30.5)	3181 (31.3)	3119 (31.5)	2970 (32.4)	1386 (32.3)
>200 000	1234 (11.5)	1206 (11.9)	1158 (11.7)	1097 (12.0)	511 (12.0)
Snoring					
None	6998 (59.0)	6626 (59.2)	6679 (61.4)	6523 (64.8)	3143 (67.3)
Nonhabitual	4053 (34.2)	3941 (35.2)	3656 (33.6)	3159 (31.4)	1375 (29.5)
Habitual	811 (6.8)	631 (5.6)	535 (4.9)	382 (3.8)	150 (3.2)

Abbreviation: BMI, body mass index.

^a^ Sex is biological sex at birth.

^b^ Race was self-selected. Other race included any race not otherwise specified.

^c^ BMI percentile was calculated as weight in kilograms divided by height in meters squared, and subsequent derivation from age- and sex-based charts from the US Centers for Disease Control and Prevention.

The proportion of male children was maintained throughout the study. The proportion of Black children decreased to 553 (11.8%) in year 5. The income category distribution was maintained, with the highest representation in the $100 000 to $200 000 per year group (ranging from 3279 participants [30.5%] in year 1 to 2970 participants [32.4%] in year 4). In year 1, 811 children (6.8%) were habitual snorers (≥3 times/week), and 4053 (34.2%) were nonhabitual snorers. Over time, the proportion of habitual snorers decreased, and by year 5, only 150 children (3.2%) were habitual snorers and 1375 (29.5%) were nonhabitual snorers.

**Figure 1.**
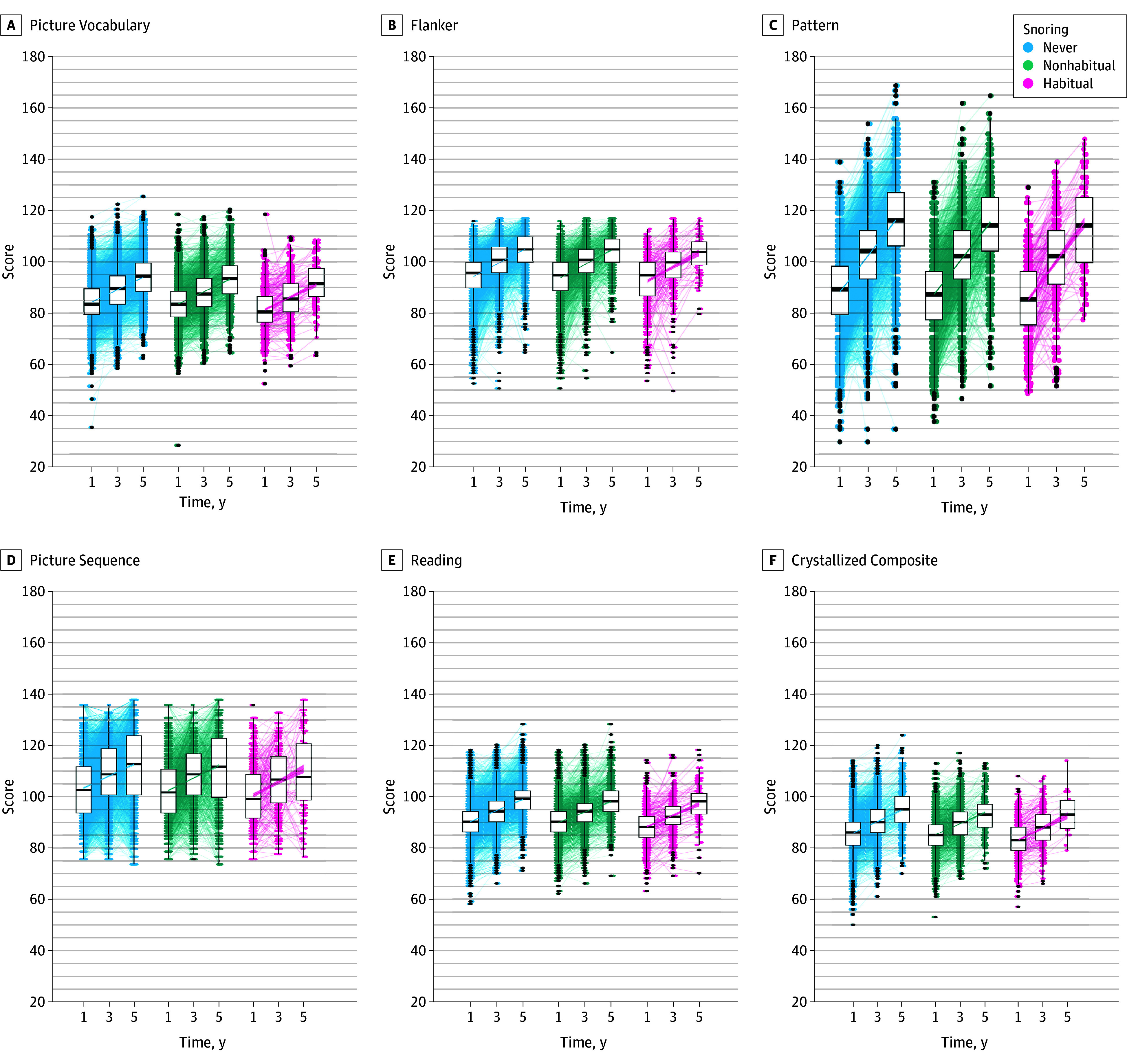
Changes in Cognitive Test Scores Among Children Stratified by the Frequency of Snoring in the Adolescent Brain Cognitive Development (ABCD) Study Cohort The 6 scores (A-F) are derived from the National Institutes of Health Toolbox (NIH-TB) cognition battery and labeled on each panel by the domain. The box plots show the data spread for each time point aggregated by the frequency of snoring (never [blue dots], nonhabitual [green dots], or habitual [pink dots]); black dots represent outliers. The line in the center of the boxes represents the median, with box edges denoting the upper and lower quartiles, and lines extending from the box representing the upper and lower extremes. Lines connect the boxplots and are presented with the associated 95% CIs for the associated fit.

**Figure 2.**
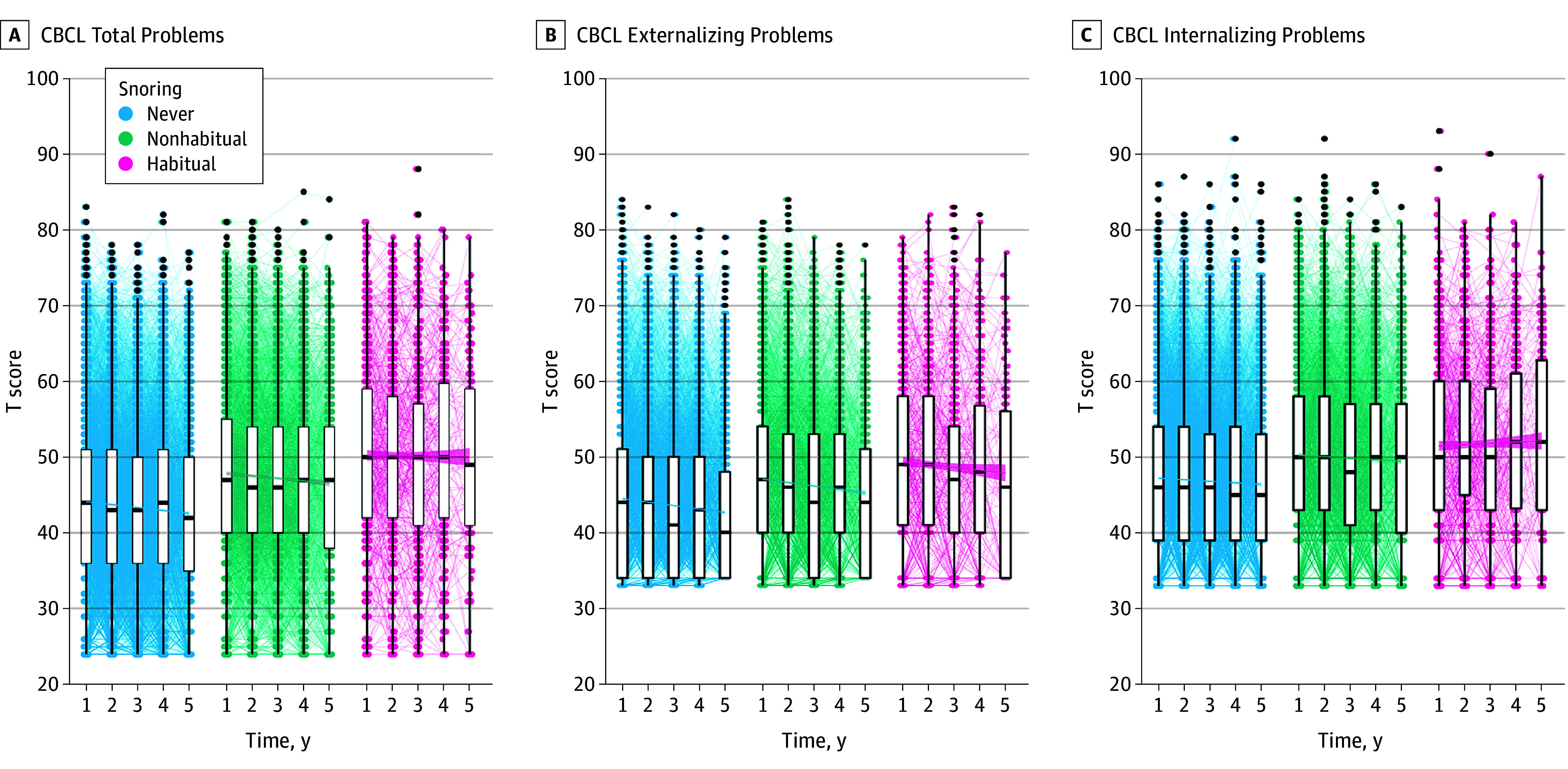
Changes in Behavior Among Children, Stratified by the Frequency of Snoring in the Adolescent Brain Cognitive Development (ABCD) Study Cohort Changes in the 3 composite scores—Child Behavior Checklist (CBCL) Total Problems (A), Externalizing Problems (B), and Internalizing Problems (C)—are shown. The box plots show the data spread for each time point aggregated by the frequency of snoring (never [blue dots], nonhabitual [green dots], or habitual [pink dots]); black dots represent outliers. The line in the center of the boxes represents the median, with box edges denoting the upper and lower quartiles, and lines extending from the box representing the upper and lower extremes. Lines connect the boxplots and are presented with the associated 95% CIs for the associated fit.

Figure 1 shows the expected age-related increases in 6 of the 10 NIH-TB cognitive test scores; the increases were similar across children stratified by the frequency of snoring. Similarly, the changes in behavior (CBCL Total, Externalizing Problems, and Internalizing Problems T scores) are shown in Figure 2; trends over time were again similar across groups. eFigure 3 in Supplement 1 shows this information for each CBCL component separately. eTable 1 in Supplement 1 shows the mean (95% CI) NIH-TB and CBCL domain and composite scores over all 5 years, highlighting that these scores were within the normal range.^32^ NIH-TB scores increased while CBCL T scores decreased over time, indicating improved cognitive performance and decreased problem behaviors.

**Figure 3.**
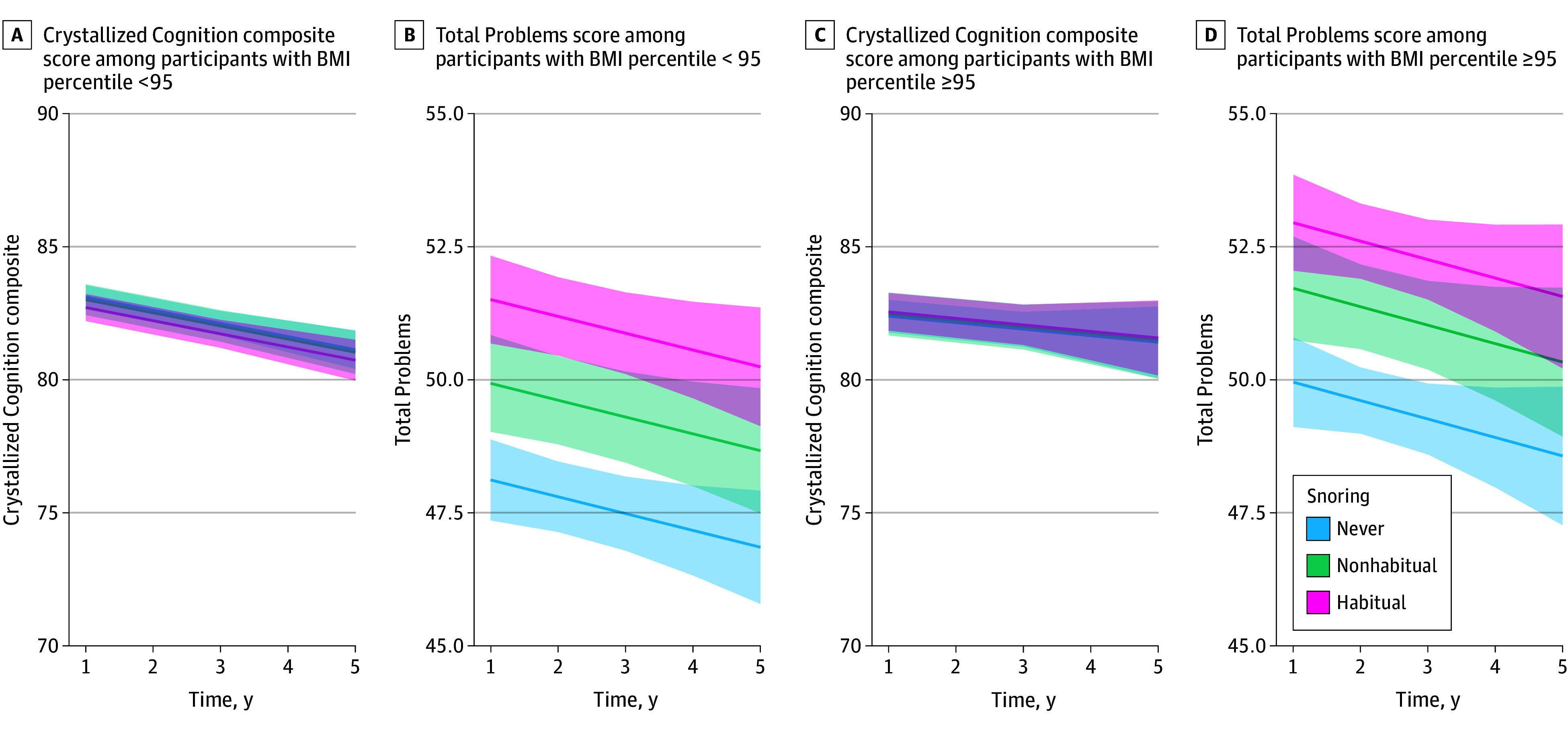
Marginal Changes in Composite Cognition and Problem Behaviors Stratified by the Frequency of Snoring and Obesity in the Adolescent Brain Cognitive Development (ABCD) Study Cohort A, marginal changes in the Crystallized Cognition composite score from the National Institutes of Health Toolbox (NIH-TB) over time (higher is better), stratified by snoring frequency in children. B, Total Problems (higher is worse) from the Child Behavior Checklist (CBCL) over time. Panels A and B are from children whose body mass index (BMI) was less than the 95th percentile. Panels C and D are respective scores in children with a BMI percentile of 95 or greater. Marginal effects were derived from linear mixed-effects regression models after controlling for age, biological sex at birth, self-selected race, total pretax household income, type of assessment (in-person, remote, or hybrid), and participant identification nested within the site as a random intercept.

Figure 3A shows the marginal changes in the NIH-TB Crystallized Composite test scores over time stratified by the frequency of snoring and obesity derived from the linear mixed-effects regression models in children without obesity after controlling for age, biological sex at birth, self-selected race, recruitment site, type of assessment (in-person, remote, or hybrid), and participant ID as a random-effect nested within site. Figure 3B shows the marginal changes in total CBCL scores over time. Figure 3C and D similarly show the corresponding marginal changes in children with obesity. The changes in NIH-TB Crystallized Composite score among habitually snoring children appeared similar to nonsnoring and non–habitually snoring children, even when stratified by obesity. However, there was a significant difference in CBCL Total Problems for children grouped by snoring frequency. eFigure 4 in Supplement 1 shows marginal changes for all NIH-TB tasks. eTables 2 to 7 in Supplement 1 show the results of the linear mixed-effects regression models fitted to all NIH-TB scores, again stratified by obesity. The fit characteristics were similar for all NIH-TB domains. For example, age (β = 0.22; 95% CI, 0.20 to 0.23; *P* < .001) and income (β = 9.17; 95% CI, 8.58 to 9.77; *P* < .001) had discernible positive associations with the Crystallized Cognition composite score among children without obesity. Negative associations with NIH-TB scores were observed for time point (β = −0.49; 95% CI, −0.69 to −0.30; *P* < .001). Race-based differences were also observed (Black: β = −4.86; 95% CI, −5.34 to −4.38; *P* < .001; other race: β = −1.37 95% CI, −1.84 to −0.90; *P* < .001). No significant interactions between snoring and time were identified. The magnitudes of these coefficients and the results of the hypothesis tests were similar for children with obesity.

eFigure 5 and eFigure 6 in Supplement 1 show the stratified marginal changes for CBCL components stratified by obesity and overall Internalizing Problems and Externalizing Problems over time. These results were similar to the negative associations of snoring with CBCL Total Problems (Figures 3B and 3D). eTables 8 to 18 in Supplement 1 show the results of linear mixed-effects models fitted to CBCL scores. Among children without obesity, the Total Problems score was positively associated with male sex (β = 1.46; 95% CI, 1.06 to 1.86; *P* < .001) and negatively associated with income (β = −4.87; 95% CI, −5.87 to −3.87; *P* < .001) (eTable 18 in Supplement 1). Compared with White participants, there were fewer caregiver-reported problem behaviors among Black participants (β = −2.71; 95% CI−3.39 to −2.04; *P* < .001) and other race participants (β = −2.60; 95% CI, −3.39 to −1.81; *P* < .001). Snoring was associated with a greater burden of problem behaviors in children without obesity (β = 2.40; 95% CI, 2.03 to 2.77; *P* < .001) and children with obesity (β = 3.18; 95% CI, 2.59 to 3.77; *P* < .001). These associations were replicated across all CBCL scores. Snoring and time did not show interactions.

## Discussion

Our 5-year longitudinal cohort study investigating the association of habitual snoring with cognitive and behavioral outcomes in adolescents supports a divergence between associations of snoring with cognition vs behavior. Significant associations with snoring were limited to problem behaviors; the lack of an association of snoring with cognitive test results confirms and extends the results from wave 1 of the study in preadolescents.^22^ Additionally, there were no interactions between time and snoring for either cognition or behavior, showing that the association of snoring with these outcomes did not vary over time. The proportion of snoring children declined over time. Together, these findings advance our understanding of the natural history of snoring in adolescents for caregivers and clinicians to consider when weighing treatment options for SDB, such as adenotonsillectomy.

The ABCD dataset enrolled children aged 9 to 10 years at baseline. Therefore, the longitudinal outcomes are from young adolescents, a marked departure from the studies performed in younger children.^12,33,34,35^ A plausible explanation for the absence of cognitive deficits in habitual snorers during adolescence is the adolescent brain’s inherent resilience and increasing cognitive reserve. During this developmental period, the brain undergoes substantial changes including synaptic pruning, increased myelination, and enhanced neural connectivity, which collectively may confer protection against the cognitive disruptions typically associated with poor sleep quality.^36^ The improved adaptive neuroplasticity in adolescence might enable the brain to have greater reserve capacity to compensate for intermittent hypoxia and sleep fragmentation, thus preserving cognitive functions such as attention, memory, and executive function.^37^

Consistent with previous studies,^38,39,40^ we found a positive association of total household income with cognitive test scores. Socioeconomic status (SES) and environmental factors are critical components of cognitive development and may moderate the impact of habitual snoring.^41^ The observed decrease in snoring adolescents over time could be attributed to a regression to normality or craniofacial expansion resulting in improved airflow. Adolescents from higher SES backgrounds often have access to better educational resources, health care, and overall living conditions, which can buffer the potential negative effects of SDB.^42,43^ Higher SES, such as parental education, can also result in greater cognitive reserve in children and young adolescents, which was shown to be more protective against brain injury.^44^ Our study controlled for SES, but the complex interplay between socioeconomic factors and cognitive reserve and resilience suggests that these variables may mitigate the adverse impacts of habitual snoring.

The significant association of habitual snoring with problem behaviors, as measured by the CBCL, highlights the importance of screening for snoring in adolescents. However, no significant interactions were found between time and snoring on CBCL measures, pointing to the potential stability of problem behaviors over time, which could be explained by other factors. First, CBCL contains sleep-related questions such as “trouble sleeping,” which could capture the underlying SDB.^45^ Second, caregiver concerns about their children’s sleep could result in overreporting of their problem behaviors, signaling a spurious association.^46^ Lastly, children may have had earlier insults to the developing brain due to untreated SDB, which could manifest in the form of problem behaviors later.^41^

In line with other studies, such as the Childhood Adenotonsillectomy Trial,^46^ the NIH-TB and CBCL scores in habitually snoring children were still in the normal ranges. The lack of cognitive deficits associated with habitual snoring has substantial implications for shared decision-making regarding treatment options like adenotonsillectomy. Parents and clinicians should consider the potential risks and costs associated with surgical interventions in young adolescents. The results from the current study support a more personalized approach to treatment, emphasizing the importance of addressing behavioral concerns while monitoring cognitive development.

For adolescents with SDB, it may be reasonable to counsel parents that their child’s snoring is unlikely to affect their cognitive abilities, either at the time of assessment or over the next few years. This information could support delaying or avoiding adenotonsillectomy. However, caregivers may understandably be concerned about the significant association of snoring with caregiver-reported behavioral problems. They may, therefore, opt for more aggressive treatment of SDB for behavioral reasons. Notably, however, our study did not find that snoring was associated with worsening existing behavioral problems over time. These findings provide helpful information that could assist clinicians and parents in weighing the risks and benefits of surgery vs watchful waiting to assess whether the snoring persists over time in treating pediatric SDB.

### Limitations

This study has limitations. The NIH-TB, while a robust tool for cognitive assessment, may not capture subtle cognitive impairments that could be associated with habitual snoring. More sensitive and specific cognitive tests or alternative methods like neuroimaging might be necessary to detect subclinical or more subtle impacts. Additionally, most children in our sample had an average BMI percentile, which may not fully represent the population at the highest risk for obstructive sleep apnea. Although the stratification by obesity did not demonstrate outcome differences, this could potentially limit the generalizability of our findings to populations with higher obesity rates.

Additionally, our classification of snoring into none, nonhabitual, and habitual categories might have led to misclassification or underestimation of the severity of snoring. Despite their higher cost and logistical demands, objective measures such as polysomnography provide more accurate assessments of snoring severity. However, polysomnography cannot realistically be used to address our question related to snoring frequency. Because polysomnographic parameters also do not reliably estimate outcomes such as cognition and behavior,^47^ the cost of performing polysomnography at a population level would not be justified. Relying on caregiver reports to assess snoring frequency introduces potential biases. Parents might underreport or misinterpret the severity of their child’s snoring, especially in adolescents whose sleep habits are difficult for parents to assess.^48^ That said, parent-reported snoring is associated with actual polysomnographic parameters such as the apnea hypopnea index and reduction of pharyngeal volumes.^49^ The ABCD dataset includes thousands of children across 21 sites in the US. Although our results are immediately pertinent to children in the US, generalization to other populations requires caution. Furthermore, children from wealthier families and urban areas were overrepresented, which could also impact the generalization of the results. This overrepresentation of affluent families might have led to a potential ceiling effect in cognitive measures, possibly limiting our ability to detect subtle cognitive differences associated with snoring. Additionally, our study did not have information on whether some of these children underwent adenotonsillectomy or other interventions for SDB during the 5-year study period. This limitation could potentially affect the interpretation of the longitudinal trends in snoring and associated outcomes.

## Conclusions

In this cohort study of adolescents in the ABCD Study, snoring was not associated with cognitive deficits or decline over 5 years. However, snoring was associated with a greater burden of problem behaviors, but children who snored did not exhibit significant worsening of their behavioral problems over time. These results may assist parents and clinicians in shared decision-making concerning the treatment of SDB in adolescents, especially before adenotonsillectomy, and choosing nonsurgical approaches such as watchful monitoring of their symptoms.

## Notes

Supplement 1. **eFigure 1.** Missingness in the Cognitive Test Scores and Related Covariates in the Adolescent Brain Cognitive Development (ABCD) Study **eFigure 2.** Missingness in the Problem Behavior Scores and Related Covariates in the Adolescent Brain Cognitive Development (ABCD) Study **eFigure 3.** Changes in Behavior Among Children Stratified by the Frequency of Snoring in the Adolescent Brain Cognitive Development (ABCD) Study Cohort **eTable 1.** Summary Cognitive and Behavior Scores Aggregated by Domain and Time **eFigure 4.** Marginal Changes in Domain-Specific Problem Behaviors Over Time in Children Stratified by Frequency of Snoring and Obesity **eTable 2.** Multivariable Regression Analysis of the Relationship Between Snoring and National Institutes of Health Toolbox Picture Vocabulary Test Score **eTable 3.** Multivariable Regression Analysis of the Relationship Between Snoring and National Institutes of Health Toolbox Flanker Inhibitory Control and Attention Test Score **eTable 4.** Multivariable Regression Analysis of the Relationship Between Snoring and National Institutes of Health Toolbox Pattern Comparison Processing Speed Test Score **eTable 5.** Multivariable Regression Analysis of the Relationship Between Snoring and National Institutes of Health Toolbox Picture Sequence Memory Score **eTable 6.** Multivariable Regression Analysis of the Relationship Between Snoring and National Institutes of Health Toolbox Reading Score **eTable 7.** Multivariable Regression Analysis of the Relationship Between Snoring and National Institutes of Health Toolbox Crystallized Cognition Composite Score **eFigure 5.** Marginal Changes in Composite (Internalizing and Externalizing) Problem Behaviors Over Time in Children Stratified by Frequency of Snoring and Obesity **eFigure 6.** Marginal Changes in Cognitive Test Scores Over Time in Children Stratified by Frequency of Snoring and Obesity **eTable 8.** Multivariable Regression Analysis of the Relationship Between Snoring and Child Behavior Checklist Anxious/Depressed Score **eTable 9.** Multivariable Regression Analysis of the Relationship Between Snoring and Child Behavior Checklist Withdrawn/Depressed Score **eTable 10.** Multivariable Regression Analysis of the Relationship Between Snoring and Child Behavior Checklist Somatic Problems Score **eTable 11.** Multivariable Regression Analysis of the Relationship Between Snoring and Child Behavior Checklist Social Problems Score **eTable 12.** Multivariable Regression Analysis of the Relationship Between Snoring and Child Behavior Checklist Thought Problems Score **eTable 13.** Multivariable Regression Analysis of the Relationship Between Snoring and Child Behavior Checklist Attention Problems Score **eTable 14.** Multivariable Regression Analysis of the Relationship Between Snoring and Child Behavior Checklist Attention Rule Breaking Score **eTable 15.** Multivariable Regression Analysis of the Relationship Between Snoring and Child Behavior Checklist Aggressive Behavior Score **eTable 16.** Multivariable Regression Analysis of the Relationship Between Snoring and Child Behavior Checklist Internalizing Problems Score **eTable 17.** Multivariable Regression Analysis of the Relationship Between Snoring and Child Behavior Checklist Externalizing Problems Score **eTable 18.** Multivariable Regression Analysis of the Relationship Between Snoring and Child Behavior Checklist Total Problems Score

Supplement 2.
Data Sharing Statement
