# Supplementary material for: Cognitive and Behavioral Outcomes of Snoring Among Adolescents
Source: JAMA Netw Open. 2024 Nov 8;7(11):e2444057. doi: 10.1001/jamanetworkopen.2024.44057 (PMC11549662; doi:10.1001/jamanetworkopen.2024.44057)
Supplement: Supplement 1. — eFigure 1. Missingness in the Cognitive Test Scores and Related Covariates in the Adolescent Brain Cognitive Development (ABCD) Study eFigure 2. Missingness in the Problem Behavior Scores and Related Covariates in the Adolescent Brain Cognitive Development (ABCD) Study eFigure 3. Changes in Behavior Among Children Stratified by the Frequency of Snoring in the Adolescent Brain Cognitive Development (ABCD) Study Cohort eTable 1. Summary Cognitive and Behavior Scores Aggregated by Domain and Time eFigure 4. Marginal Changes in Domain-Specific Problem Behaviors Over Time in Children Stratified by Frequency of Snoring and Obesity eTable 2. Multivariable Regression Analysis of the Relationship Between Snoring and National Institutes of Health Toolbox Picture Vocabulary Test Score eTable 3. Multivariable Regression Analysis of the Relationship Between Snoring and National Institutes of Health Toolbox Flanker Inhibitory Control and Attention Test Score eTable 4. Multivariable Regression Analysis of the Relationship Between Snoring and National Institutes of Health Toolbox Pattern Comparison Processing Speed Test Score eTable 5. Multivariable Regression Analysis of the Relationship Between Snoring and National Institutes of Health Toolbox Picture Sequence Memory Score eTable 6. Multivariable Regression Analysis of the Relationship Between Snoring and National Institutes of Health Toolbox Reading Score eTable 7. Multivariable Regression Analysis of the Relationship Between Snoring and National Institutes of Health Toolbox Crystallized Cognition Composite Score eFigure 5. Marginal Changes in Composite (Internalizing and Externalizing) Problem Behaviors Over Time in Children Stratified by Frequency of Snoring and Obesity eFigure 6. Marginal Changes in Cognitive Test Scores Over Time in Children Stratified by Frequency of Snoring and Obesity eTable 8. Multivariable Regression Analysis of the Relationship Between Snoring and Child Behavior Checklist Anxious/Depressed Score eTable [file jamanetwopen-e2444057-s001.pdf]

Supplemental Online Content

Isaiah A, Uddin S, Ernst T, Cloak C, Li D, Chang L. Cognitive and behavioral outcomes of snoring among adolescents. *JAMA Netw. Open*. 2024;7(11):e2444057. doi:10.1001/jamanetworkopen.2024.44057

eFigure 1. Missingness in the Cognitive Test Scores and Related Covariates in the Adolescent Brain Cognitive Development (ABCD) Study
eFigure 2. Missingness in the Problem Behavior Scores and Related Covariates in the Adolescent Brain Cognitive Development (ABCD) Study
eFigure 3. Changes in Behavior Among Children Stratified by the Frequency of Snoring in the Adolescent Brain Cognitive Development (ABCD) Study Cohort
eTable 1. Summary Cognitive and Behavior Scores Aggregated by Domain and Time
eFigure 4. Marginal Changes in Domain-Specific Problem Behaviors Over Time in Children Stratified by Frequency of Snoring and Obesity
eTable 2. Multivariable Regression Analysis of the Relationship Between Snoring and National Institutes of Health Toolbox Picture Vocabulary Test Score
eTable 3. Multivariable Regression Analysis of the Relationship Between Snoring and National Institutes of Health Toolbox Flanker Inhibitory Control and Attention Test Score
eTable 4. Multivariable Regression Analysis of the Relationship Between Snoring and National Institutes of Health Toolbox Patteran Comparison Processing Speed Test Score
eTable 5. Multivariable Regression Analysis of the Relationship Between Snoring and National Institutes of Health Toolbox Picture Sequence Memory Score
eTable 6. Multivariable Regression Analysis of the Relationship Between Snoring and National Institutes of Health Toolbox Reading Score
eTable 7. Multivariable Regression Analysis of the Relationship Between Snoring and National Institutes of Health Toolbox Crystallized Cognition Composite Score
eFigure 5. Marginal Changes in Composite (Internalizing and Externalizing) Problem Behaviors Over Time in Children Stratified by Frequency of Snoring and Obesity
eFigure 6. Marginal Changes in Cognitive Test Scores Over Time in Children Stratified by Frequency of Snoring and Obesity
eTable 8. Multivariable Regression Analysis of the Relationship Between Snoring and Child Behavior Checklist Anxious/Depressed Score
eTable 9. Multivariable Regression Analysis of the Relationship Between Snoring and Child Behavior Checklist Withdrawn/Depressed Score
eTable 10. Multivariable Regression Analysis of the Relationship Between Snoring and Child Behavior Checklist Somatic Problems Score
eTable 11. Multivariable Regression Analysis of the Relationship Between Snoring and Child Behavior Checklist Social Problems Score
eTable 12. Multivariable Regression Analysis of the Relationship Between Snoring and Child Behavior Checklist Thought Problems Score
eTable 13. Multivariable Regression Analysis of the Relationship Between Snoring and Child Behavior Checklist Attention Problems Score
eTable 14. Multivariable Regression Analysis of the Relationship Between Snoring and Child Behavior Checklist Attention Rule Breaking Score
eTable 15. Multivariable Regression Analysis of the Relationship Between Snoring and Child Behavior Checklist Aggressive Behavior Score

© 2024 Isaiah A et al. *JAMA Network Open.*

eTable 16. Multivariable Regression Analysis of the Relationship Between Snoring and Child Behavior Checklist Internalizing Problems Score
eTable 17. Multivariable Regression Analysis of the Relationship Between Snoring and Child Behavior Checklist Externalizing Problems Score
eTable 18. Multivariable Regression Analysis of the Relationship Between Snoring and Child Behavior Checklist Total Problems Score

This supplemental material has been provided by the authors to give readers additional information about their work.

© 2024 Isaiah A et al. *JAMA Network Open.*

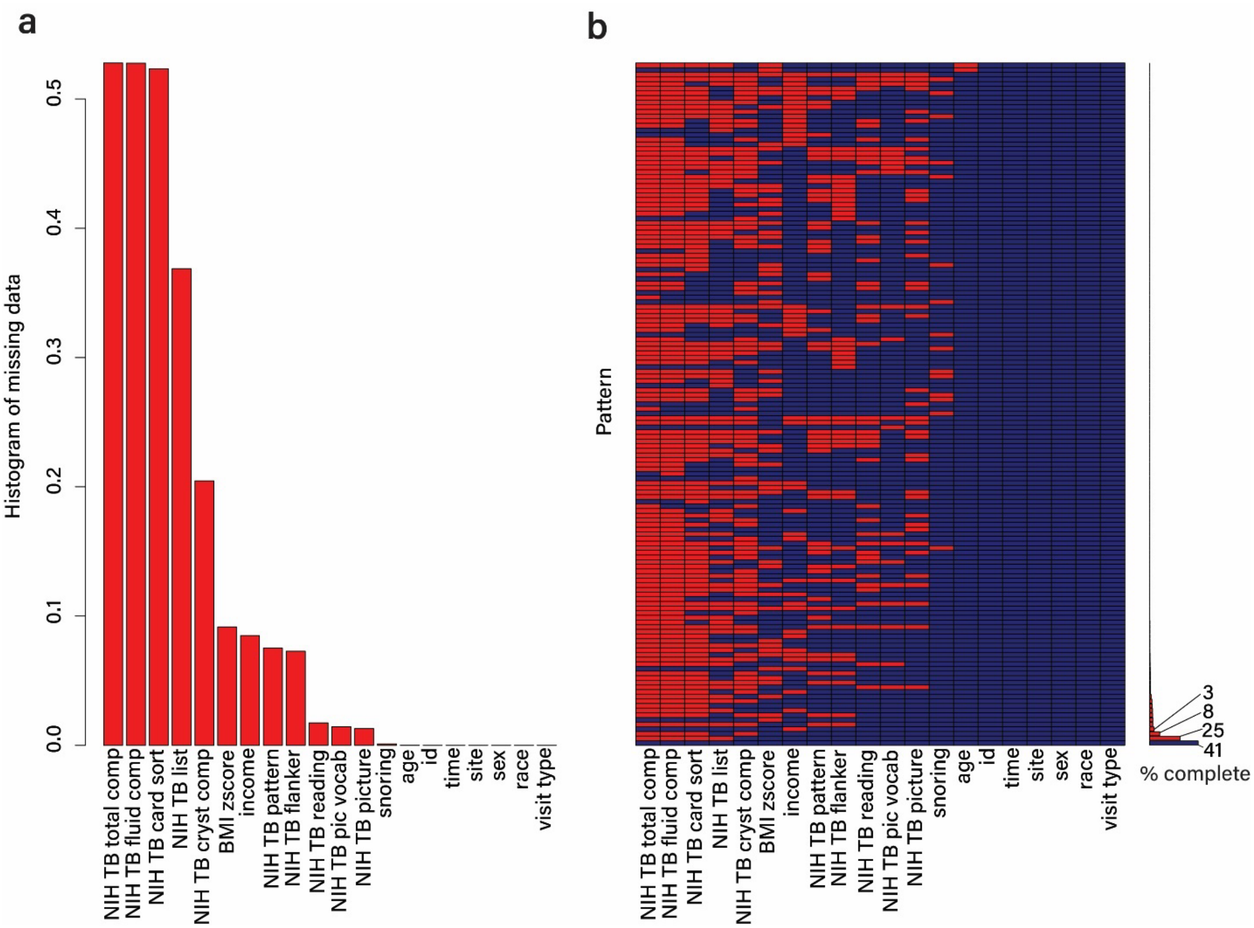

eFigure 1. Missingness in the cognitive test scores and related covariates in the Adolescent Brain Cognitive Development (ABCD) study. (a) shows a histogram of missing data, with over 50% of the data for the National Institutes of Health Toolbox (NIH-TB) missing in the domains of total composite, fluid composite, and card sort tests. (b) shows the distribution of missingness across combinations of different variables, with red indicating any missingness. The plot to the right shows the proportion of the data with specific patterns of missingness indicated by the red tiles.

© 2024 Isaiah A et al. *JAMA Network Open.*

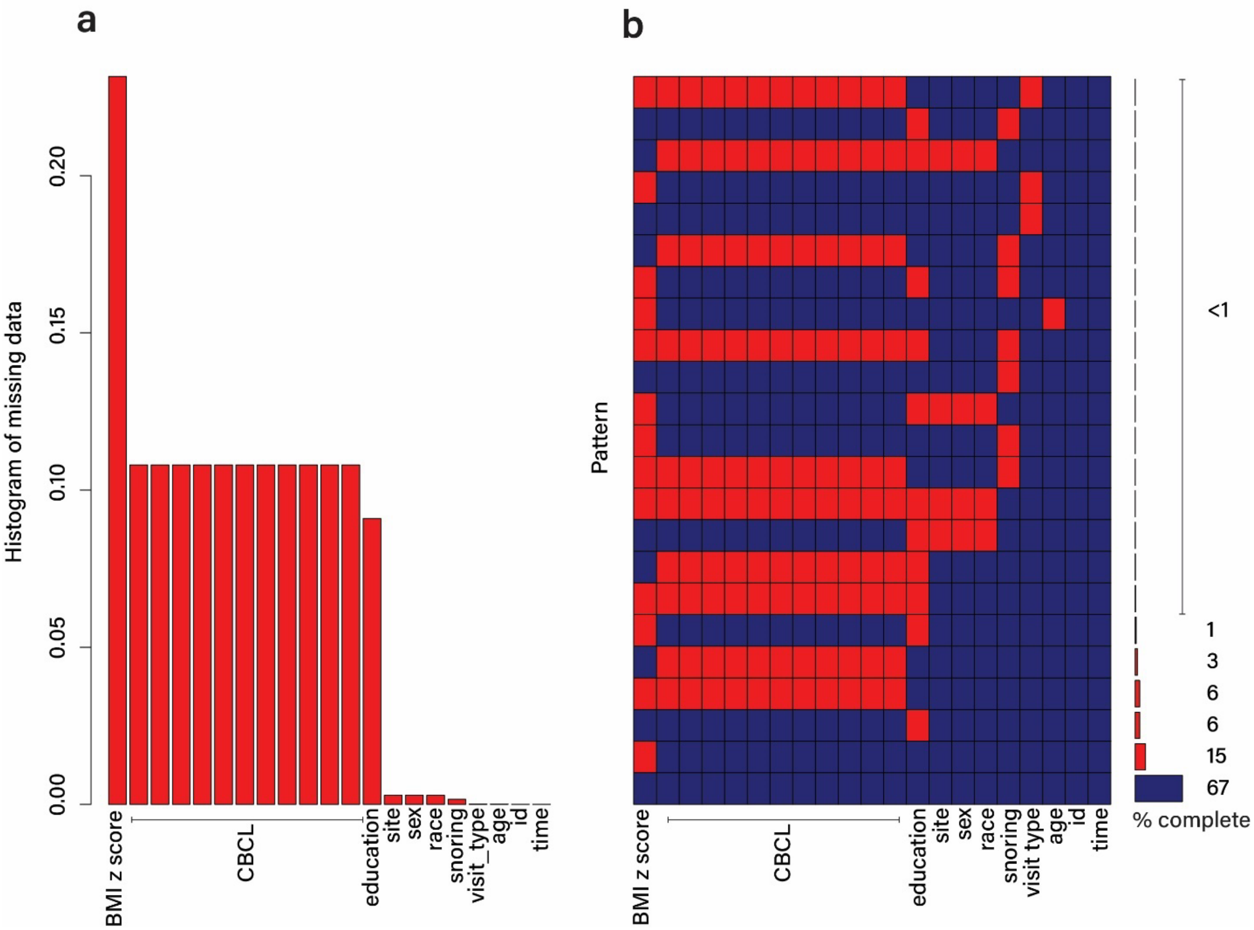

eFigure 2. Missingness in the problem behavior scores and related covariates in the Adolescent Brain Cognitive Development (ABCD) study. (a) shows a histogram of missing data, with the most missing being the body mass index (BMI) z score. The histogram also shows acceptable missingness (~10%) for Child Behavior Checklist (CBCL) scores across its subscales. (b) shows the distribution of missingness across combinations of different variables, with red indicating missingness. The plot to the right shows the proportion of the data with specific patterns of missingness indicated by the red tiles.

© 2024 Isaiah A et al. *JAMA Network Open.*

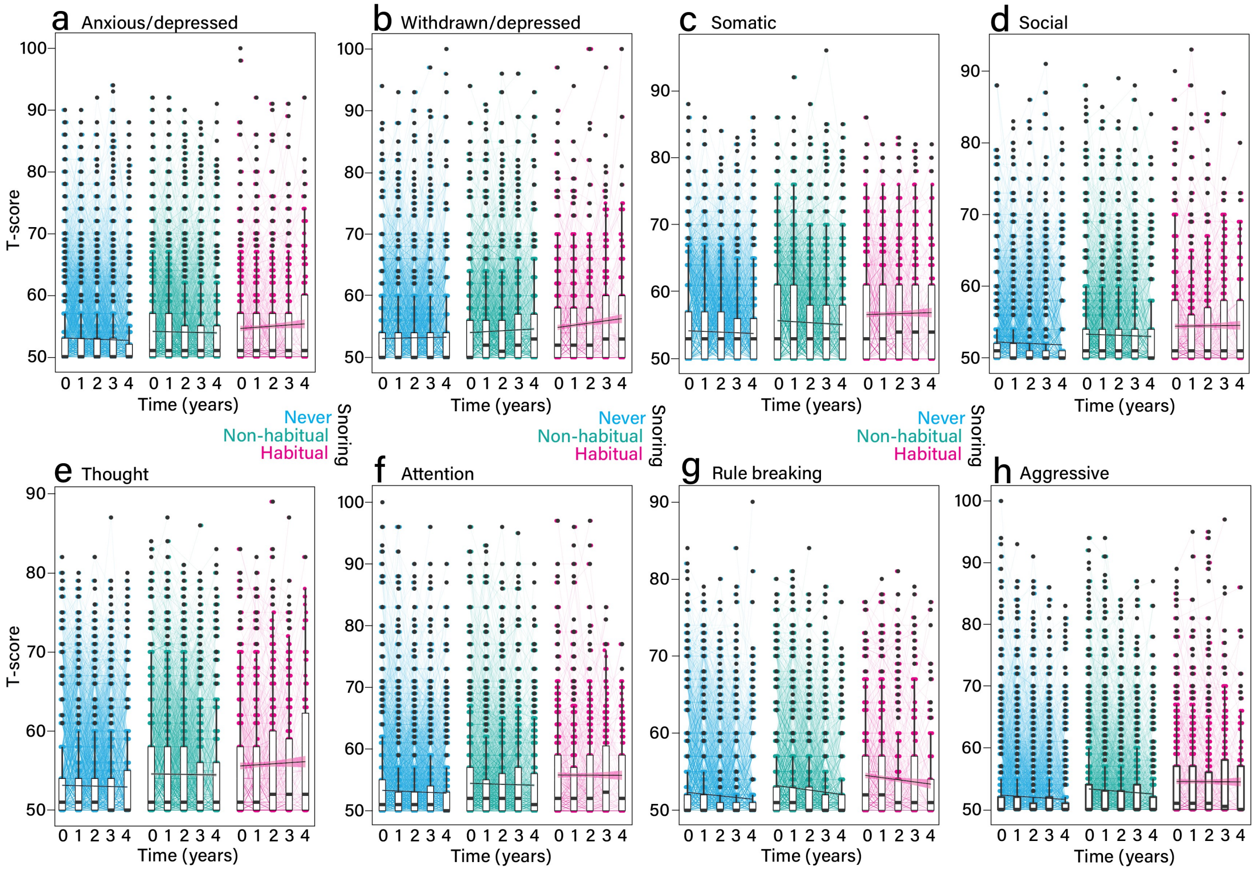

eFigure 3. Changes in behavior among children stratified by the frequency of snoring in the Adolescent Brain Cognitive Development (ABCD) study cohort. The eight scores are derived from the Child Behavior Checklist (CBCL). The x-axis represents the time (baseline, 1-year, 2-year, 3-year, and 4-year follow-up). The y-axis represents the T score. The boxplots show the data spread for each time point aggregated by the frequency of snoring (never, non-habitual, or habitual). Lines connect the boxplots with associated confidence intervals.

© 2024 Isaiah A et al. *JAMA Network Open.*

Test	Domain	Year 1	Year 2	Year 3	Year 4	Year 5
NIH-TB, mean score (95% CI)	Picture vocabulary	84.5 (84.3, 84.6)	NA	89.0 (88.8, 89.2)	NA	94.4 (94.1, 94.7)
	Flanker	94.0 (93.8, 94.2)	NA	100.2 (100.0, 100.3)	NA	104.4 (104.1, 104.6)
	Pattern	88.1 (87.8, 88.3)	NA	103.6 (103.3, 103.9)	NA	115.7 (115.1, 116.3)
	Picture sequence	102.8 (102.6, 103.0)	NA	109.0 (108.7, 109.2)	NA	111.6 (111.2, 112.1)
	Reading	90.9 (90.7, 91.0)	NA	94.9 (94.7, 95.0)	NA	99.7 (99.4, 99.9)
	Crystallized composite	86.4 (86.2, 86.5)	NA	90.9 (90.8, 91.1)	NA	95.3 (94.6, 95.9)
CBCL, mean (95% CI)	Anxious/depressed	53.5 (53.4, 53.6)	53.5 (53.4, 53.6)	53.2 (53.1, 53.3)	53.26 (53.12, 53.39)	53.1 (53.0, 53.3)
	Withdrawn/depressed	53.5 (53.4, 53.6)	53.7 (53.6, 53.8)	53.5 (53.3, 53.6)	53.80 (53.67, 53.93)	53.8 (53.7, 54.0)
	Somatic	54.9 (54.8, 55.0)	54.8 (54.6, 54.9)	54.6 (54.5, 54.7)	54.48 (54.35, 54.61)	54.3 (54.1, 54.4)
	Social	52.8 (52.7, 52.9)	52.6 (52.5, 52.7)	52.6 (52.5, 52.7)	52.46 (52.35, 52.56)	52.2 (52.1, 52.4)
	Thought	53.8 (53.7, 53.9)	53.8 (53.7, 53.9)	53.6 (53.5, 53.7)	53.58 (53.46, 53.71)	53.5 (53.4, 53.7)
	Attention	53.9 (53.8, 54.0)	53.7 (53.6, 53.8)	53.5 (53.4, 53.6)	53.62 (53.49, 53.74)	53.3 (53.1, 53.5)
	Rule break	52.8 (52.7, 52.9)	52.6 (52.5, 52.7)	52.1 (52.0, 52.2)	51.94 (51.85, 52.02)	51.8 (51.7, 51.9)
	Aggressive	52.8 (52.7, 52.9)	52.6 (52.5, 52.7)	52.4 (52.3, 52.5)	52.33(52.22, 52.43)	51.9 (51.8, 52.0)
	Internal problems	48.5 (48.3, 48.7)	48.6 (48.4, 48.8)	47.8 (47.6, 48.0)	47.94 (47.70, 48.17)	47.4 (47.1, 47.7)
	External problems	45.7 (45.5, 45.9)	45.2 (45.0, 45.4)	44.5 (44.3, 44.7)	44.44 (44.22, 44.65)	43.4 (43.2, 43.7)
	Total problems	45.9 (45.7, 46.1)	45.5 (45.2, 45.7)	44.8 (44.5, 45.0)	44.82 (44.56, 45.07)	43.8 (43.5, 44.2)

eTable 1. Summary cognitive and behavior scores aggregated by domain and time. Mean (95% confidence intervals) are shown for the National Institutes of Health Toolbox (NIH-TB) and Child Behavior Checklist (CBCL), listed by the timing of data collection (years 1 to 5) and the domains listed.

© 2024 Isaiah A et al. *JAMA Network Open.*

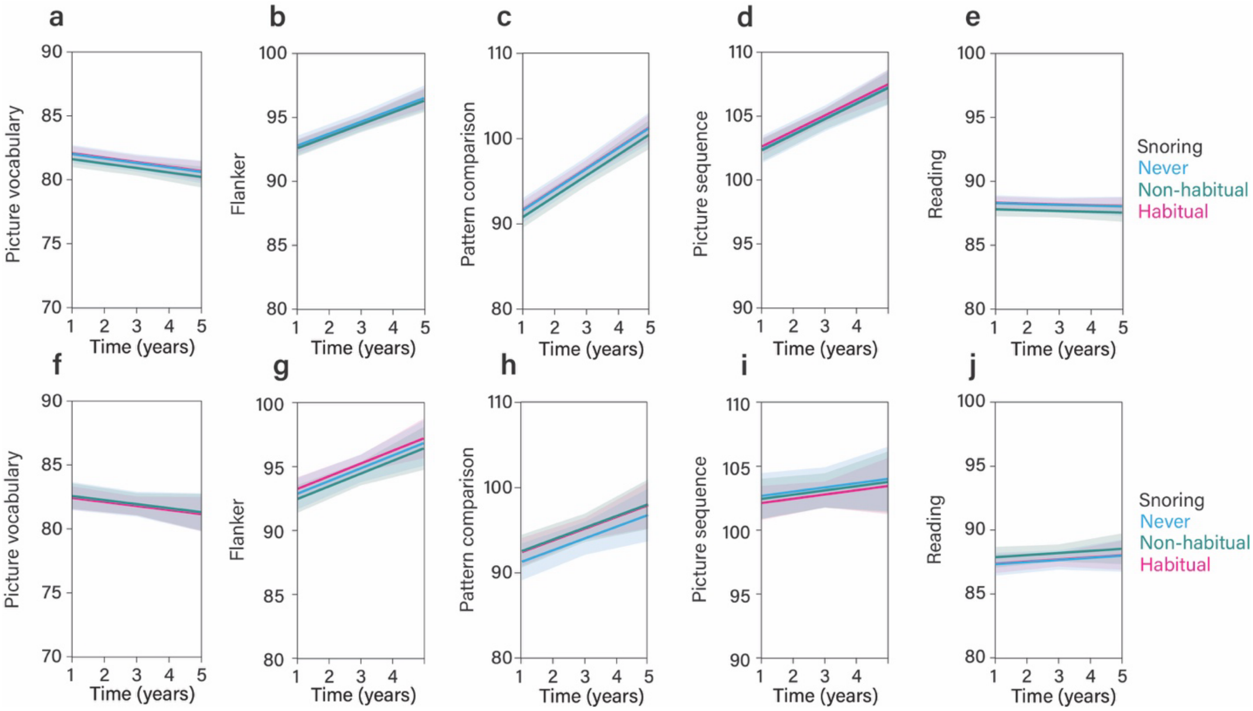

eFigure 4. Marginal changes in cognitive test scores over time in children stratified by frequency of snoring and obesity. (a-e) Plots represent the changes in composite marginal scores from the National Institutes of Health Toolbox (NIH-TB) over time (higher is better), stratified by snoring frequency (a-h) in non-obese patients, defined as body mass index (BMI) percentile < 95, while (f-j) show scores in children who are deemed obese by BMI ≥ 95. Marginal effects are derived from linear mixed-effects regression models after controlling for the fixed effects of age, biological sex at birth, self-selected race, type of assessment (in-person, remote, or hybrid), and participant ID nested within the recruitment site as a random effect. Snoring had a main effect on CBCL scores over time. However, there were no interaction effects related to snoring *x* time.

© 2024 Isaiah A et al. *JAMA Network Open.*

Fixed effects						
	Non-obese			Obese		
Predictors	Estimates	CI	P	Estimates	CI	P
(Intercept)	59.27 ***	57.40 to 61.14	**<0.001**	59.76 ***	58.05 to 61.46	**<0.001**
Snoring	-0.32	-0.66 to 0.01	0.054	-0.18	-0.45 to 0.08	0.181
Age	0.21 ***	0.19 to 0.22	**<0.001**	0.20 ***	0.19 to 0.22	**<0.001**
Male	0.25	-0.02 to 0.52	0.065	0.26 *	0.01 to 0.50	**0.038**
Race (Black)	-4.59 ***	-5.04 to -4.13	**<0.001**	-4.33 ***	-4.72 to -3.94	**<0.001**
Race (Other)	-2.49 ***	-3.02 to -1.96	**<0.001**	-2.47 ***	-2.94 to -1.99	**<0.001**
Income	9.77 ***	9.10 to 10.45	**<0.001**	9.53 ***	8.95 to 10.10	**<0.001**
Time	-0.35 **	-0.56 to -0.13	**0.002**	-0.32 **	-0.52 to -0.13	**0.001**
Visit type (Remote)	-2.05	-5.34 to 1.24	0.222	0.18	-0.23 to 0.60	0.389
Visit type (Hybrid)	0.20	-0.08 to 0.47	0.160	0.09	-0.15 to 0.34	0.443
**Random Effects**						
σ^2	18.77				17.92	
$\tau_{00\ id:site}$	31.61				31.17	
$\tau_{00\ site}$	0.84				1.44	
ICC	0.63				0.65	
N_{id}	9173				2374	
N_{site}	22				22	
Observations	17784				3885	
Marginal R^2 / Conditional R^2	0.341 / 0.758				0.334 / 0.764	

eTable 2. Multivariable regression analysis of the relationship between snoring and National Institutes of Health Toolbox Picture Vocabulary Test score. Linear mixed-effects regression model contained the following fixed effects: age in months, biological sex at birth (comparison = female), race as self-selected by the primary caregiver (comparison = White), income in 10 classes as described in the text as an ordered factor, and time (comparison = baseline). Results are stratified by children grouped by whether they were obese or not, as defined by a body mass index (BMI) percentile ≥ 95. Random effects included the participant ID nested within the recruitment site ID. The coefficients of each predictor, their 95% confidence intervals, and the associated P values are provided. Fit is described by adjusted R^2, of which the marginal R^2 considers only the variance of the fixed effects, while the conditional R^2 considers both the fixed and random effects. The intraclass correlation coefficient (ICC) computes the proportion of the variance explained by the grouping structure in the population. τ_{00} indicates how much groups or subjects differ from each other, while the residual variance σ^2 indicates the within-subject variance. The subscripted N represents the number of children (id) and the number of sites (site).

© 2024 Isaiah A et al. *JAMA Network Open.*

Fixed effects						
	Non-obese			Obese		
Predictors	Estimates	CI	P	Estimates	CI	P
(Intercept)	75.07 ***	73.08 to 77.07	**<0.001**	77.76 ***	73.38 to 82.14	**<0.001**
Snoring	-0.13	-0.54 to 0.28	0.529	-0.56	-1.22 to 0.10	0.099
Age	0.15 ***	0.13 to 0.16	**<0.001**	0.12 ***	0.08 to 0.16	**<0.001**
Male	0.33 *	0.05 to 0.61	**0.020**	0.82 *	0.18 to 1.45	**0.011**
Race (Black)	-2.14 ***	-2.62 to -1.66	**<0.001**	-2.41 ***	-3.20 to -1.61	**<0.001**
Race (Other)	0.14	-0.41 to 0.70	0.615	-0.59	-1.66 to 0.48	0.279
Income	5.29 ***	4.58 to 6.00	**<0.001**	4.41 ***	3.14 to 5.69	**<0.001**
Time	0.92 ***	0.68 to 1.16	**<0.001**	0.99 ***	0.45 to 1.52	**<0.001**
Visit type (Remote)	5.27	-0.02 to 10.55	0.051	6.13	-9.10 to 21.36	0.430
Visit type (Hybrid)	-0.36	-0.76 to 0.03	0.074	-0.55	-1.42 to 0.32	0.216
**Random Effects**						
σ^2	37.73			42.51		
$\tau_{00\ id:site}$	24.62			30.10		
$\tau_{00\ site}$	0.55			0.26		
ICC	0.40			0.42		
N_{id}	9164			2324		
N_{site}	22			22		
Observations	17326			3756		
Marginal R^2 / Conditional R^2	0.237 / 0.542			0.210 / 0.539		

eTable 3. Multivariable regression analysis of the relationship between snoring and National Institutes of Health Toolbox Flanker Inhibitory Control and Attention Test score. Linear mixed-effects regression model contained the following fixed effects: age in months, biological sex at birth (comparison = female), race as self-selected by the primary caregiver (comparison = White), income in 10 classes as described in the text as an ordered factor, and time (comparison = baseline). Results are stratified by children grouped by whether they were obese or not, as defined by a body mass index (BMI) percentile ≥ 95. Random effects included the participant ID nested within the recruitment site ID. The coefficients of each predictor, their 95% confidence intervals, and the associated P values are provided. Fit is described by adjusted R^2, of which the marginal R^2 considers only the variance of the fixed effects, while the conditional R^2 considers both the fixed and random effects. The intraclass correlation coefficient (ICC) computes the proportion of the variance explained by the grouping structure in the population. τ_{00} indicates how much groups or subjects differ from each other, while the residual variance σ^2 indicates the within-subject variance. The subscripted N represents the number of children (id) and the number of sites (site).

© 2024 Isaiah A et al. *JAMA Network Open.*

Fixed effects						
	**Non-obese**			**Obese**		
Predictors	Estimates	CI	P	Estimates	CI	P
(Intercept)	39.10 ***	35.42 to 42.77	**<0.001**	34.90 ***	27.36 to 42.43	**<0.001**
Snoring	-0.60	-1.34 to 0.13	0.108	0.08	-1.04 to 1.21	0.888
Age	0.40 ***	0.37 to 0.43	**<0.001**	0.44 ***	0.37 to 0.51	**<0.001**
Male	-2.31 ***	-2.83 to -1.80	**<0.001**	-2.10 ***	-3.18 to -1.02	**<0.001**
Race (Black)	-3.03 ***	-3.92 to -2.15	**<0.001**	-2.79 ***	-4.18 to -1.40	**<0.001**
Race (Other)	-0.28	-1.30 to 0.74	0.586	-0.51	-2.37 to 1.35	0.591
Income	4.99 ***	3.69 to 6.29	**<0.001**	3.92 ***	1.72 to 6.11	**<0.001**
Time	2.41 ***	1.97 to 2.85	**<0.001**	1.37 **	0.45 to 2.29	**0.003**
Visit type (Remote)	6.59	-2.82 to 16.01	0.170	14.20	-11.60 to 40.00	0.281
Visit type (Hybrid)	-1.26 ***	-1.97 to -0.56	**<0.001**	-0.88	-2.37 to 0.60	0.242
**Random Effects**						
σ^2	117.69			121.16		
$\tau_{00\ id:site}$	87.60			88.84		
$\tau_{00\ site}$	2.83			2.28		
ICC	0.43			0.43		
N_{id}	9157			2322		
N_{site}	22			22		
Observations	17285			3742		
Marginal R^2 / Conditional R^2	0.369 / 0.643			0.358 / 0.634		

eTable 4. Multivariable regression analysis of the relationship between snoring and National Institutes of Health Toolbox Pattern Comparison Processing Speed test score. Linear mixed-effects regression model contained the following fixed effects: age in months, biological sex at birth (comparison = female), race as self-selected by the primary caregiver (comparison = White), income in 10 classes as described in the text as an ordered factor, and time (comparison = baseline). Results are stratified by children grouped by whether they were obese or not, as defined by a body mass index (BMI) percentile ≥ 95. Random effects included the participant ID nested within the recruitment site ID. The coefficients of each predictor, their 95% confidence intervals, and the associated P values are provided. Fit is described by adjusted R^2, of which the marginal R^2 considers only the variance of the fixed effects, while the conditional R^2 considers both the fixed and random effects. The intraclass correlation coefficient (ICC) computes the proportion of the variance explained by the grouping structure in the population. τ_{00} indicates how much groups or subjects differ from each other, while the residual variance σ^2 indicates the within-subject variance. The subscripted N represents the number of children (id) and the number of sites (site).

© 2024 Isaiah A et al. *JAMA Network Open.*

Fixed effects						
	Non-obese			Obese		
Predictors	Estimates	CI	P	Estimates	CI	P
(Intercept)	85.71 ***	82.78 to 88.65	**<0.001**	80.47 ***	74.35 to 86.60	**<0.001**
Snoring	-0.21	-0.83 to 0.42	0.517	0.22	-0.72 to 1.17	0.643
Age	0.14 ***	0.11 to 0.17	**<0.001**	0.19 ***	0.13 to 0.24	**<0.001**
Male	-1.47 ***	-1.89 to -1.05	**<0.001**	-1.41 **	-2.28 to -0.53	**0.002**
Race (Black)	-5.69 ***	-6.40 to -4.98	**<0.001**	-3.99 ***	-5.12 to -2.87	**<0.001**
Race (Other)	-0.71	-1.52 to 0.11	0.091	-1.00	-2.50 to 0.50	0.191
Income	6.12 ***	5.07 to 7.18	**<0.001**	4.79 ***	3.02 to 6.57	**<0.001**
Time	1.22 ***	0.86 to 1.57	**<0.001**	0.33	-0.43 to 1.09	0.391
Visit type (Remote)	0.04	-6.75 to 6.83	0.991	4.32	-18.29 to 26.93	0.708
Visit type (Hybrid)	-3.37 ***	-3.93 to -2.80	**<0.001**	-3.17 ***	-4.37 to -1.97	**<0.001**
**Random Effects**						
σ^2	90.24			99.48		
$\tau_{00\ id:site}$	55.30			49.32		
$\tau_{00\ site}$	0.14			1.11		
ICC	0.38			0.34		
N_{id}	9171			2376		
N_{site}	22			22		
Observations	17823			3891		
Marginal R^2 / Conditional R^2	0.141 / 0.468			0.123 / 0.418		

eTable 5. Multivariable regression analysis of the relationship between snoring and National Institutes of Health Toolbox Picture sequence memory score. Linear mixed-effects regression model contained the following fixed effects: age in months, biological sex at birth (comparison = female), race as self-selected by the primary caregiver (comparison = White), income in 10 classes as described in the text as an ordered factor, and time (comparison = baseline). Results are stratified by children grouped by whether they were obese or not, as defined by a body mass index (BMI) percentile ≥ 95. Random effects included the participant ID nested within the recruitment site ID. The coefficients of each predictor, their 95% confidence intervals, and the associated P values are provided. Fit is described by adjusted R^2, of which the marginal R^2 considers only the variance of the fixed effects, while the conditional R^2 considers both the fixed and random effects. The intraclass correlation coefficient (ICC) computes the proportion of the variance explained by the grouping structure in the population. τ_{00} indicates how much groups or subjects differ from each other, while the residual variance σ^2 indicates the within-subject variance. The subscripted N represents the number of children (id) and the number of sites (site).

© 2024 Isaiah A et al. *JAMA Network Open.*

Fixed effects						
	Non-obese			Obese		
Predictors	Estimates	CI	P	Estimates	CI	P
(Intercept)	70.79 ***	69.15 to 72.44	**<0.001**	71.77 ***	68.42 to 75.13	**<0.001**
Snoring	-0.37 **	-0.64 to -0.09	**0.009**	0.35	-0.05 to 0.75	0.084
Age	0.16 ***	0.15 to 0.18	**<0.001**	0.15 ***	0.12 to 0.18	**<0.001**
Male	-0.12	-0.35 to 0.12	0.338	0.10	-0.39 to 0.60	0.681
Race (Black)	-2.28 ***	-2.68 to -1.88	**<0.001**	-1.64 ***	-2.28 to -1.00	**<0.001**
Race (Other)	-0.03	-0.50 to 0.44	0.891	-0.32	-1.18 to 0.53	0.461
Income	7.52 ***	6.92 to 8.11	**<0.001**	8.09 ***	7.08 to 9.09	**<0.001**
Time	-0.06	-0.25 to 0.12	0.503	0.16	-0.22 to 0.55	0.408
Visit type (Remote)	0.07	-2.58 to 2.71	0.960	-0.72	-8.82 to 7.39	0.862
Visit type (Hybrid)	0.27 *	0.05 to 0.49	**0.015**	0.23	-0.21 to 0.67	0.308
**Random Effects**						
σ^2	11.80			9.69		
$\tau_{00\ id:site}$	26.67			30.41		
$\tau_{00\ site}$	0.66			0.51		
ICC	0.70			0.76		
N_{id}	9172			2371		
N_{site}	22			22		
Observations	17741			3874		
Marginal R^2 / Conditional R^2	0.286 / 0.785			0.309 / 0.835		

eTable 6. Multivariable regression analysis of the relationship between snoring and National Institutes of Health Toolbox Reading score. Linear mixed-effects regression model contained the following fixed effects: age in months, biological sex at birth (comparison = female), race as self-selected by the primary caregiver (comparison = White), income in 10 classes as described in the text as an ordered factor, and time (comparison = baseline). Results are stratified by children grouped by whether they were obese or not, as defined by a body mass index (BMI) percentile ≥ 95. Random effects included the participant ID nested within the recruitment site ID. The coefficients of each predictor, their 95% confidence intervals, and the associated P values are provided. Fit is described by adjusted R^2, of which the marginal R^2 considers only the variance of the fixed effects, while the conditional R^2 considers both the fixed and random effects. The intraclass correlation coefficient (ICC) computes the proportion of the variance explained by the grouping structure in the population. τ_{00} indicates how much groups or subjects differ from each other, while the residual variance σ^2 indicates the within-subject variance. The subscripted N represents the number of children (id) and the number of sites (site).

© 2024 Isaiah A et al. *JAMA Network Open.*

Fixed effects						
	Non-obese			Obese		
Predictors	Estimates	CI	P	Estimates	CI	P
(Intercept)	60.18 ***	58.49 to 61.86	**<0.001**	62.22 ***	58.68 to 65.75	**<0.001**
Snoring	-0.28 *	-0.55 to -0.00	**0.047**	0.12	-0.30 to 0.54	0.588
Age	0.22 ***	0.20 to 0.23	**<0.001**	0.19 ***	0.16 to 0.23	**<0.001**
Male	0.09	-0.15 to 0.32	0.478	0.16	-0.35 to 0.67	0.530
Race (Black)	-3.54 ***	-3.95 to -3.14	**<0.001**	-2.77 ***	-3.42 to -2.12	**<0.001**
Race (Other)	-1.37 ***	-1.84 to -0.90	**<0.001**	-1.52 ***	-2.39 to -0.65	**0.001**
Income	9.17 ***	8.58 to 9.77	**<0.001**	8.75 ***	7.73 to 9.78	**<0.001**
Time	-0.49 ***	-0.69 to -0.30	**<0.001**	-0.25	-0.65 to 0.16	0.240
Visit type (Remote)	-0.81	-4.31 to 2.69	0.648	-0.53	-7.90 to 6.83	0.887
Visit type (Hybrid)	-1.94 ***	-2.48 to -1.41	**<0.001**	-1.89 ***	-2.95 to -0.82	**0.001**
**Random Effects**						
σ^2	9.10			7.87		
$\tau_{00\ id:site}$	27.15			29.02		
$\tau_{00\ site}$	0.64			0.45		
ICC	0.75			0.79		
N_{id}	9118			2125		
N_{site}	22			22		
Observations	14977			3098		
Marginal R^2 / Conditional R^2	0.333 / 0.835			0.337 / 0.860		

eTable 7. Multivariable regression analysis of the relationship between snoring and National Institutes of Health Toolbox Crystallized cognition composite score. Linear mixed-effects regression model contained the following fixed effects: age in months, biological sex at birth (comparison = female), race as self-selected by the primary caregiver (comparison = White), income in 10 classes as described in the text as an ordered factor, and time (comparison = baseline). Results are stratified by children grouped by whether they were obese or not, as defined by a body mass index (BMI) percentile ≥ 95. Random effects included the participant ID nested within the recruitment site ID. The coefficients of each predictor, their 95% confidence intervals, and the associated P values are provided. Fit is described by adjusted R^2, of which the marginal R^2 considers only the variance of the fixed effects, while the conditional R^2 considers both the fixed and random effects. The intraclass correlation coefficient (ICC) computes the proportion of the variance explained by the grouping structure in the population. τ_{00} indicates how much groups or subjects differ from each other, while the residual variance σ^2 indicates the within-subject variance. The subscripted N represents the number of children (id) and the number of sites (site).

© 2024 Isaiah A et al. *JAMA Network Open.*

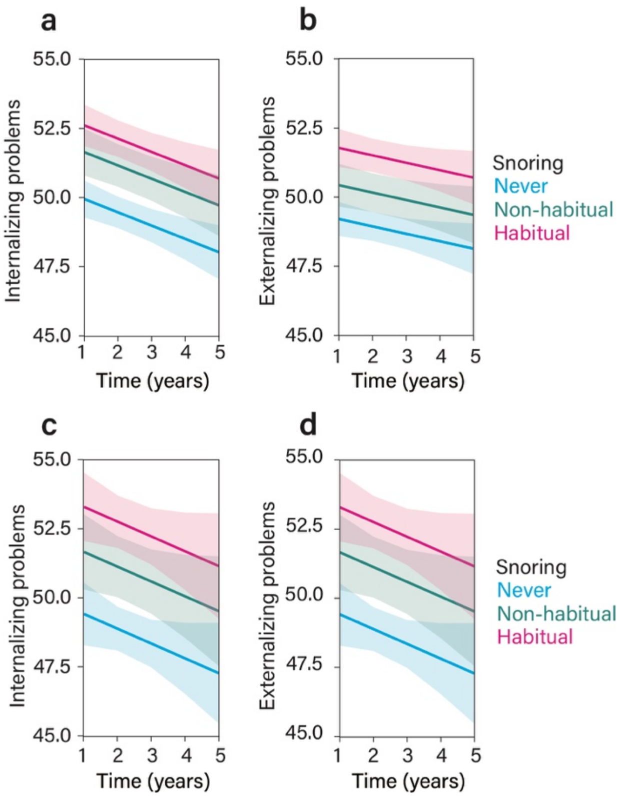

eFigure 5. Marginal changes in composite (internalizing and externalizing) problem behaviors over time in children stratified by frequency of snoring and obesity. (a-b) show scores from Child Behavior Checklist (CBCL) Internalizing and Externalizing problems scores over time (higher is worse), stratified by snoring frequency in non-obese patients, defined as body mass index (BMI) percentile < 95, while (c-d) show corresponding scores in children who are deemed obese by BMI ≥ 95. Marginal effects are derived from linear mixed-effects regression models after controlling for the fixed effects of age, biological sex at birth, self-selected race, type of assessment (in-person, remote, or hybrid), and participant ID nested within the recruitment site as a random effect. Snoring had a main effect on CBCL scores over time. However, there were no interaction effects related to snoring *x* time.

© 2024 Isaiah A et al. *JAMA Network Open.*

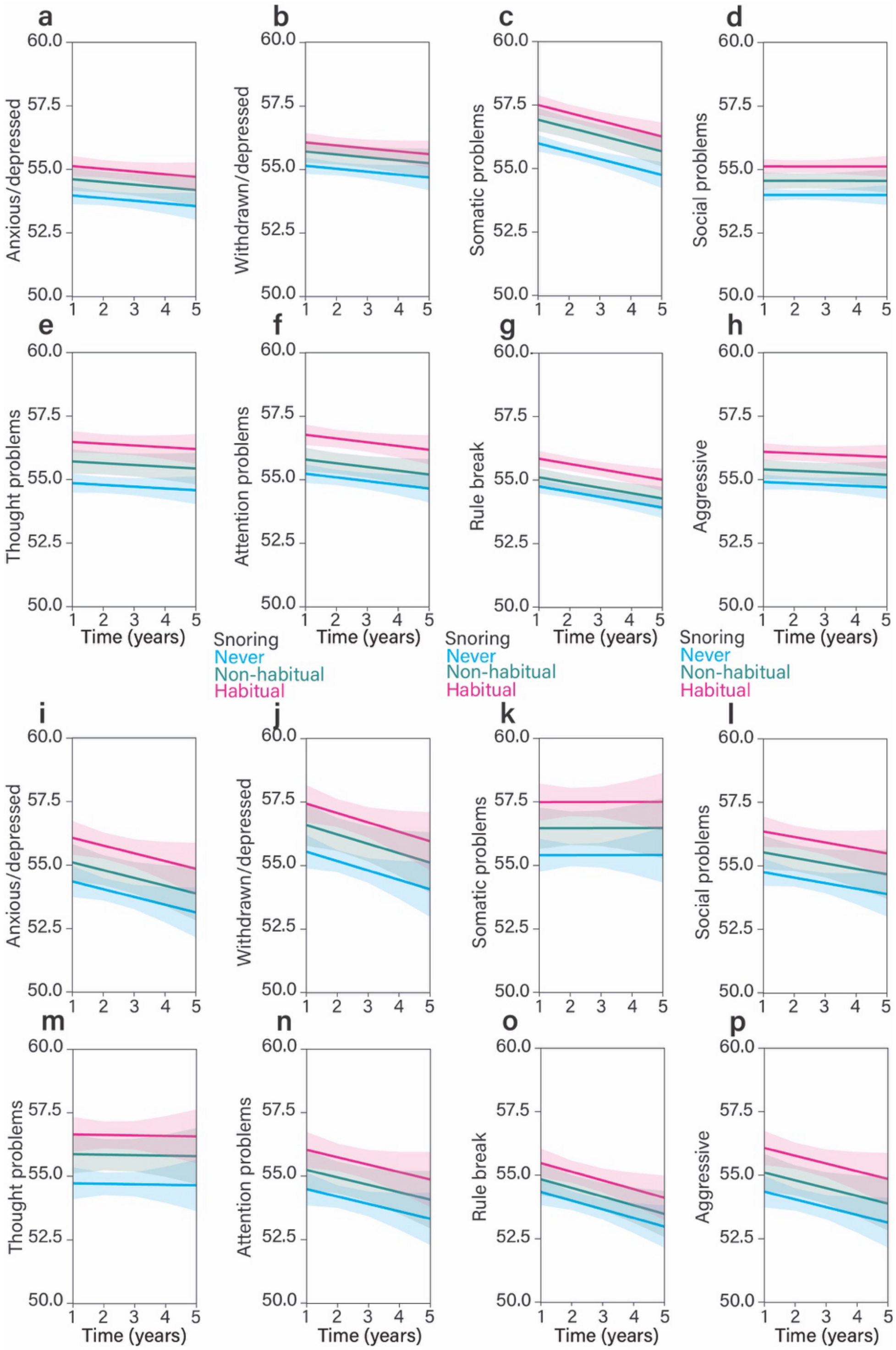

eFigure 6. Marginal changes in problem behaviors over time in children stratified by frequency of snoring and obesity. (a-h) show scores from the Child Behavior Checklist (CBCL) over time (higher is worse), stratified by snoring frequency in non-obese patients, defined as body mass index (BMI) percentile < 95, while (i-p) show scores in children who are deemed obese by BMI ≥ 95. Marginal effects are derived from linear mixed-effects regression models after controlling for the fixed effects of age, biological sex at birth, self-selected race, type of assessment (in-person, remote, or hybrid), and participant ID nested within the recruitment site as a random effect. Snoring had a main effect on CBCL scores over time. However, there were no interaction effects related to snoring *x* time.

© 2024 Isaiah A et al. *JAMA Network Open.*

Fixed effects						
	Non-obese			Obese		
Predictors	Estimates	CI	P	Estimates	CI	P
(Intercept)	53.77 ***	52.35 to 55.20	**<0.001**	52.44 ***	49.42 to 55.46	**<0.001**
Snoring	0.81 ***	0.59 to 1.04	**<0.001**	1.09 ***	0.74 to 1.44	**<0.001**
Age	0.00	-0.01 to 0.02	0.544	0.02	-0.01 to 0.05	0.147
Male	0.27 *	0.06 to 0.48	**0.010**	0.34	-0.10 to 0.78	0.130
Race (Black)	-1.65 ***	-2.00 to -1.30	**<0.001**	-2.15 ***	-2.72 to -1.58	**<0.001**
Race (Other)	-1.01 ***	-1.42 to -0.60	**<0.001**	-1.04 **	-1.80 to -0.28	**0.007**
Income	-1.15 ***	-1.67 to -0.63	**<0.001**	-1.17 **	-2.06 to -0.28	**0.010**
Time	-0.11	-0.27 to 0.06	0.211	-0.29	-0.65 to 0.06	0.106
Visit type (Remote)	-0.65	-2.49 to 1.18	0.484	-1.53	-7.61 to 4.55	0.621
Visit type (Hybrid)	0.05	-0.18 to 0.27	0.684	-0.04	-0.54 to 0.46	0.868
**Random Effects**						
σ^2	13.11			14.85		
$\tau_{00\ id:site}$	20.91			23.36		
$\tau_{00\ site}$	0.19			0.33		
ICC	0.62			0.61		
N_{id}	9278			2529		
N_{site}	22			22		
Observations	26665			5800		
Marginal R^2 / Conditional R^2	0.015 / 0.622			0.030 / 0.626		

eTable 8. Multivariable regression analysis of the relationship between snoring and Child Behavior Checklist Anxious/depressed score. Linear mixed-effects regression model contained the following fixed effects: age in months, biological sex at birth (comparison = female), race as self-selected by the primary caregiver (comparison = White), income in 10 classes as described in the text as an ordered factor, and time (comparison = baseline). Results are stratified by children grouped by whether they were obese or not, as defined by a body mass index (BMI) percentile ≥ 95. Random effects included the participant ID nested within the recruitment site ID. The coefficients of each predictor, their 95% confidence intervals, and the associated P values are provided. Fit is described by adjusted R^2, of which the marginal R^2 considers only the variance of the fixed effects, while the conditional R^2 considers both the fixed and random effects. The intraclass correlation coefficient (ICC) computes the proportion of the variance explained by the grouping structure in the population. τ_{00} indicates how much groups or subjects differ from each other, while the residual variance σ^2 indicates the within-subject variance. The subscripted N represents the number of children (id) and the number of sites (site).

© 2024 Isaiah A et al. *JAMA Network Open.*

Fixed effects						
	Non-obese			Obese		
Predictors	Estimates	CI	P	Estimates	CI	P
(Intercept)	52.67 ***	51.34 to 53.99	**<0.001**	51.14 ***	47.97 to 54.30	**<0.001**
Snoring	0.65 ***	0.43 to 0.87	**<0.001**	1.34 ***	0.95 to 1.73	**<0.001**
Age	0.01	-0.00 to 0.02	0.071	0.04 *	0.01 to 0.06	**0.017**
Male	0.93 ***	0.74 to 1.13	**<0.001**	0.86 ***	0.40 to 1.32	**<0.001**
Race (Black)	-0.89 ***	-1.21 to -0.57	**<0.001**	-1.78 ***	-2.37 to -1.19	**<0.001**
Race (Other)	-0.49 *	-0.87 to -0.12	**0.010**	-1.02 *	-1.80 to -0.23	**0.011**
Income	-2.64 ***	-3.12 to -2.16	**<0.001**	-2.12 ***	-3.05 to -1.19	**<0.001**
Time	-0.12	-0.27 to 0.04	0.145	-0.37	-0.74 to 0.01	0.054
Visit type (Remote)	-1.19	-3.03 to 0.65	0.204	-5.86	-12.72 to 1.01	0.095
Visit type (Hybrid)	0.36 **	0.13 to 0.59	**0.002**	0.39	-0.17 to 0.95	0.170
**Random Effects**						
σ^2	13.47			19.15		
$\tau_{00\ id:site}$	16.83			24.14		
$\tau_{00\ site}$	0.14			0.21		
ICC	0.56			0.56		
N_{id}	9278			2529		
N_{site}	22			22		
Observations	26665			5800		
Marginal R^2 / Conditional R^2	0.027 / 0.570			0.033 / 0.574		

eTable 9. Multivariable regression analysis of the relationship between snoring and Child Behavior Checklist Withdrawn/depressed score. Linear mixed-effects regression model contained the following fixed effects: age in months, biological sex at birth (comparison = female), race as self-selected by the primary caregiver (comparison = White), income in 10 classes as described in the text as an ordered factor, and time (comparison = baseline). Results are stratified by children grouped by whether they were obese or not, as defined by a body mass index (BMI) percentile ≥ 95. Random effects included the participant ID nested within the recruitment site ID. The coefficients of each predictor, their 95% confidence intervals, and the associated P values are provided. Fit is described by adjusted R^2, of which the marginal R^2 considers only the variance of the fixed effects, while the conditional R^2 considers both the fixed and random effects. The intraclass correlation coefficient (ICC) computes the proportion of the variance explained by the grouping structure in the population. τ_{00} indicates how much groups or subjects differ from each other, while the residual variance σ^2 indicates the within-subject variance. The subscripted N represents the number of children (id) and the number of sites (site).

© 2024 Isaiah A et al. *JAMA Network Open.*

Fixed effects						
	Non-obese			Obese		
Predictors	Estimates	CI	P	Estimates	CI	P
(Intercept)	54.24 ***	52.88 to 55.60	**<0.001**	56.65 ***	53.52 to 59.78	**<0.001**
Snoring	1.07 ***	0.83 to 1.30	**<0.001**	1.48 ***	1.09 to 1.87	**<0.001**
Age	0.02 **	0.01 to 0.03	**0.005**	0.00	-0.03 to 0.03	0.929
Male	-0.55 ***	-0.74 to -0.35	**<0.001**	-0.23	-0.69 to 0.22	0.319
Race (Black)	-1.45 ***	-1.78 to -1.11	**<0.001**	-1.68 ***	-2.27 to -1.10	**<0.001**
Race (Other)	-1.06 ***	-1.44 to -0.67	**<0.001**	-1.25 **	-2.03 to -0.48	**0.002**
Income	-1.79 ***	-2.28 to -1.30	**<0.001**	-1.14 *	-2.05 to -0.22	**0.015**
Time	-0.31 ***	-0.47 to -0.15	**<0.001**	0.00	-0.37 to 0.37	0.991
Visit type (Remote)	-0.66	-2.64 to 1.31	0.511	0.05	-6.94 to 7.04	0.989
Visit type (Hybrid)	-0.09	-0.34 to 0.15	0.443	-0.38	-0.95 to 0.19	0.186
**Random Effects**						
σ^2	15.74			19.97		
$\tau_{00\ id:site}$	16.97			22.73		
$\tau_{00\ site}$	0.14			0.24		
ICC	0.52			0.53		
N_{id}	9278			2529		
N_{site}	22			22		
Observations	26665			5800		
Marginal R^2 / Conditional R^2	0.022 / 0.531			0.028 / 0.548		

eTable 10. Multivariable regression analysis of the relationship between snoring and Child Behavior Checklist Somatic problems score. Linear mixed-effects regression model contained the following fixed effects: age in months, biological sex at birth (comparison = female), race as self-selected by the primary caregiver (comparison = White), income in 10 classes as described in the text as an ordered factor, and time (comparison = baseline). Results are stratified by children grouped by whether they were obese or not, as defined by a body mass index (BMI) percentile ≥ 95. Random effects included the participant ID nested within the recruitment site ID. The coefficients of each predictor, their 95% confidence intervals, and the associated P values are provided. Fit is described by adjusted R^2, of which the marginal R^2 considers only the variance of the fixed effects, while the conditional R^2 considers both the fixed and random effects. The intraclass correlation coefficient (ICC) computes the proportion of the variance explained by the grouping structure in the population. τ_{00} indicates how much groups or subjects differ from each other, while the residual variance σ^2 indicates the within-subject variance. The subscripted N represents the number of children (id) and the number of sites (site).

© 2024 Isaiah A et al. *JAMA Network Open.*

Fixed effects						
	Non-obese			Obese		
Predictors	Estimates	CI	P	Estimates	CI	P
(Intercept)	53.54 ***	52.47 to 54.60	**<0.001**	53.00 ***	50.39 to 55.61	**<0.001**
Snoring	0.79 ***	0.63 to 0.96	**<0.001**	1.13 ***	0.83 to 1.44	**<0.001**
Age	-0.00	-0.01 to 0.01	0.724	0.01	-0.01 to 0.03	0.399
Male	0.50 ***	0.34 to 0.66	**<0.001**	0.55 **	0.17 to 0.93	**0.005**
Race (Black)	-0.55 ***	-0.81 to -0.28	**<0.001**	-0.83 ***	-1.32 to -0.34	**0.001**
Race (Other)	-0.72 ***	-1.03 to -0.41	**<0.001**	-1.05 **	-1.70 to -0.39	**0.002**
Income	-2.66 ***	-3.05 to -2.27	**<0.001**	-2.35 ***	-3.12 to -1.58	**<0.001**
Time	-0.00	-0.13 to 0.12	0.990	-0.22	-0.52 to 0.09	0.169
Visit type (Remote)	-0.59	-1.95 to 0.76	0.393	-0.15	-5.39 to 5.09	0.956
Visit type (Hybrid)	-0.16	-0.32 to 0.01	0.064	0.05	-0.38 to 0.48	0.828
**Random Effects**						
σ^2	7.13			11.02		
$\tau_{00\ id:site}$	12.14			17.84		
$\tau_{00\ site}$	0.06			0.12		
ICC	0.63			0.62		
N_{id}	9278			2529		
N_{site}	22			22		
Observations	26665			5800		
Marginal R^2 / Conditional R^2	0.033 / 0.643			0.031 / 0.631		

eTable 11. Multivariable regression analysis of the relationship between snoring and Child Behavior Checklist Social problems score. Linear mixed-effects regression model contained the following fixed effects: age in months, biological sex at birth (comparison = female), race as self-selected by the primary caregiver (comparison = White), income in 10 classes as described in the text as an ordered factor, and time (comparison = baseline). Results are stratified by children grouped by whether they were obese or not, as defined by a body mass index (BMI) percentile ≥ 95. Random effects included the participant ID nested within the recruitment site ID. The coefficients of each predictor, their 95% confidence intervals, and the associated P values are provided. Fit is described by adjusted R^2, of which the marginal R^2 considers only the variance of the fixed effects, while the conditional R^2 considers both the fixed and random effects. The intraclass correlation coefficient (ICC) computes the proportion of the variance explained by the grouping structure in the population. τ_{00} indicates how much groups or subjects differ from each other, while the residual variance σ^2 indicates the within-subject variance. The subscripted N represents the number of children (id) and the number of sites (site).

© 2024 Isaiah A et al. *JAMA Network Open.*

Fixed effects						
	Non-obese			Obese		
Predictors	Estimates	CI	Estimates	CI	Estimates	CI
(Intercept)	53.90 ***	52.49 to 55.30	**<0.001**	53.00 ***	50.39 to 55.61	**<0.001**
Snoring	1.15 ***	0.93 to 1.37	**<0.001**	1.13 ***	0.83 to 1.44	**<0.001**
Age	0.01	-0.01 to 0.02	0.339	0.01	-0.01 to 0.03	0.399
Male	0.61 ***	0.41 to 0.81	**<0.001**	0.55 **	0.17 to 0.93	**0.005**
Race (Black)	-1.18 ***	-1.53 to -0.83	**<0.001**	-0.83 ***	-1.32 to -0.34	**0.001**
Race (Other)	-1.13 ***	-1.54 to -0.73	**<0.001**	-1.05 **	-1.70 to -0.39	**0.002**
Income	-2.03 ***	-2.54 to -1.52	**<0.001**	-2.35 ***	-3.12 to -1.58	**<0.001**
Time	-0.07	-0.23 to 0.09	0.399	-0.22	-0.52 to 0.09	0.169
Visit type (Remote)	-0.25	-2.07 to 1.56	0.785	-0.15	-5.39 to 5.09	0.956
Visit type (Hybrid)	-0.01	-0.23 to 0.21	0.906	0.05	-0.38 to 0.48	0.828
**Random Effects**						
σ^2	12.84			11.02		
$\tau_{00\ id:site}$	19.98			17.84		
$\tau_{00\ site}$	0.31			0.12		
ICC	0.61			0.62		
N_{id}	9278			2529		
N_{site}	22			22		
Observations	26665			5800		
Marginal R^2 / Conditional R^2	0.021 / 0.621			0.031 / 0.631		

eTable 12. Multivariable regression analysis of the relationship between snoring and Child Behavior Checklist Thought problems score. Linear mixed-effects regression model contained the following fixed effects: age in months, biological sex at birth (comparison = female), race as self-selected by the primary caregiver (comparison = White), income in 10 classes as described in the text as an ordered factor, and time (comparison = baseline). Results are stratified by children grouped by whether they were obese or not, as defined by a body mass index (BMI) percentile ≥ 95. Random effects included the participant ID nested within the recruitment site ID. The coefficients of each predictor, their 95% confidence intervals, and the associated P values are provided. Fit is described by adjusted R^2, of which the marginal R^2 considers only the variance of the fixed effects, while the conditional R^2 considers both the fixed and random effects. The intraclass correlation coefficient (ICC) computes the proportion of the variance explained by the grouping structure in the population. τ_{00} indicates how much groups or subjects differ from each other, while the residual variance σ^2 indicates the within-subject variance. The subscripted N represents the number of children (id) and the number of sites (site).

© 2024 Isaiah A et al. *JAMA Network Open.*

Fixed effects						
	Non-obese			Obese		
Predictors	Estimates	CI	Estimates	CI	Estimates	CI
(Intercept)	54.71 ***	53.27 to 56.15	**<0.001**	53.54 ***	50.65 to 56.42	**<0.001**
Snoring	1.08 ***	0.88 to 1.29	**<0.001**	1.48 ***	1.17 to 1.80	**<0.001**
Age	0.00	-0.01 to 0.01	0.879	0.01	-0.01 to 0.04	0.379
Male	0.55 ***	0.34 to 0.77	**<0.001**	0.10	-0.33 to 0.53	0.640
Race (Black)	-0.41 *	-0.77 to -0.05	**0.027**	-0.80 **	-1.35 to -0.26	**0.004**
Race (Other)	-1.02 ***	-1.44 to -0.60	**<0.001**	-0.92 *	-1.65 to -0.19	**0.014**
Income	-2.45 ***	-2.98 to -1.91	**<0.001**	-1.98 ***	-2.84 to -1.12	**<0.001**
Time	-0.15	-0.31 to 0.02	0.081	-0.25	-0.58 to 0.09	0.150
Visit type (Remote)	-1.12	-2.73 to 0.50	0.174	-6.80 *	-12.09 to -1.51	**0.012**
Visit type (Hybrid)	0.30 **	0.11 to 0.50	**0.002**	0.44	-0.00 to 0.87	0.050
**Random Effects**						
σ^2	9.88			11.12		
$\tau_{00\ id:site}$	23.78			23.39		
$\tau_{00\ site}$	0.26			0.22		
ICC	0.71			0.68		
N_{id}	9278			2529		
N_{site}	22			22		
Observations	26665			5800		
Marginal R^2 / Conditional R^2	0.023 / 0.715			0.027 / 0.688		

eTable 13. Multivariable regression analysis of the relationship between snoring and Child Behavior Checklist Attention problems score. Linear mixed-effects regression model contained the following fixed effects: age in months, biological sex at birth (comparison = female), race as self-selected by the primary caregiver (comparison = White), income in 10 classes as described in the text as an ordered factor, and time (comparison = baseline). Results are stratified by children grouped by whether they were obese or not, as defined by a body mass index (BMI) percentile ≥ 95. Random effects included the participant ID nested within the recruitment site ID. The coefficients of each predictor, their 95% confidence intervals, and the associated P values are provided. Fit is described by adjusted R^2, of which the marginal R^2 considers only the variance of the fixed effects, while the conditional R^2 considers both the fixed and random effects. The intraclass correlation coefficient (ICC) computes the proportion of the variance explained by the grouping structure in the population. τ_{00} indicates how much groups or subjects differ from each other, while the residual variance σ^2 indicates the within-subject variance. The subscripted N represents the number of children (id) and the number of sites (site).

© 2024 Isaiah A et al. *JAMA Network Open.*

Fixed effects						
	Non-obese			Obese		
Predictors	Estimates	CI	Estimates	CI	Estimates	CI
(Intercept)	53.95 ***	52.89 to 55.01	**<0.001**	52.97 ***	50.55 to 55.40	**<0.001**
Snoring	0.77 ***	0.61 to 0.94	**<0.001**	0.80 ***	0.52 to 1.08	**<0.001**
Age	-0.00	-0.01 to 0.01	0.723	0.01	-0.01 to 0.03	0.408
Male	0.32 ***	0.17 to 0.48	**<0.001**	0.13	-0.22 to 0.49	0.458
Race (Black)	0.19	-0.07 to 0.45	0.148	-0.31	-0.77 to 0.15	0.186
Race (Other)	-0.55 ***	-0.86 to -0.25	**<0.001**	-0.89 **	-1.50 to -0.27	**0.005**
Income	-3.27 ***	-3.66 to -2.89	**<0.001**	-2.24 ***	-2.96 to -1.52	**<0.001**
Time	-0.21 ***	-0.33 to -0.08	**0.001**	-0.34 *	-0.62 to -0.06	**0.019**
Visit type (Remote)	0.02	-1.34 to 1.38	0.979	-1.37	-6.15 to 3.42	0.576
Visit type (Hybrid)	0.29 ***	0.12 to 0.45	**0.001**	0.10	-0.30 to 0.49	0.635
**Random Effects**						
σ^2	7.19			9.17		
$\tau_{00\ id:site}$	11.54			15.37		
$\tau_{00\ site}$	0.15			0.25		
ICC	0.62			0.63		
N_{id}	9278			2529		
N_{site}	22			22		
Observations	26665			5800		
Marginal R^2 / Conditional R^2	0.047 / 0.637			0.028 / 0.641		

eTable 14. Multivariable regression analysis of the relationship between snoring and Child Behavior Checklist Attention Rule breaking score. Linear mixed-effects regression model contained the following fixed effects: age in months, biological sex at birth (comparison = female), race as self-selected by the primary caregiver (comparison = White), income in 10 classes as described in the text as an ordered factor, and time (comparison = baseline). Results are stratified by children grouped by whether they were obese or not, as defined by a body mass index (BMI) percentile ≥ 95. Random effects included the participant ID nested within the recruitment site ID. The coefficients of each predictor, their 95% confidence intervals, and the associated P values are provided. Fit is described by adjusted R^2, of which the marginal R^2 considers only the variance of the fixed effects, while the conditional R^2 considers both the fixed and random effects. The intraclass correlation coefficient (ICC) computes the proportion of the variance explained by the grouping structure in the population. τ_{00} indicates how much groups or subjects differ from each other, while the residual variance σ^2 indicates the within-subject variance. The subscripted N represents the number of children (id) and the number of sites (site).

© 2024 Isaiah A et al. *JAMA Network Open.*

Fixed effects						
	Non-obese			Obese		
Predictors	Estimates	CI	Estimates	CI	Estimates	CI
(Intercept)	54.34 ***	53.12 to 55.57	**<0.001**	53.62 ***	50.73 to 56.50	**<0.001**
Snoring	0.84 ***	0.66 to 1.02	**<0.001**	1.22 ***	0.90 to 1.53	**<0.001**
Age	-0.01	-0.02 to 0.00	0.240	0.00	-0.02 to 0.03	0.722
Male	0.70 ***	0.52 to 0.88	**<0.001**	0.68 **	0.25 to 1.10	**0.002**
Race (Black)	-0.64 ***	-0.95 to -0.33	**<0.001**	-0.95 ***	-1.50 to -0.40	**0.001**
Race (Other)	-0.88 ***	-1.24 to -0.53	**<0.001**	-1.07 **	-1.81 to -0.34	**0.004**
Income	-3.35 ***	-3.80 to -2.89	**<0.001**	-2.03 ***	-2.89 to -1.17	**<0.001**
Time	-0.05	-0.19 to 0.09	0.470	-0.31	-0.64 to 0.03	0.073
Visit type (Remote)	-0.17	-1.62 to 1.27	0.814	-5.16	-10.41 to 0.10	0.054
Visit type (Hybrid)	0.04	-0.13 to 0.22	0.630	0.19	-0.24 to 0.62	0.391
**Random Effects**						
σ^2	7.97			10.97		
$\tau_{00\ id:site}$	16.78			23.23		
$\tau_{00\ site}$	0.14			0.37		
ICC	0.68			0.68		
N_{id}	9278			2529		
N_{site}	22			22		
Observations	26665			5800		
Marginal R^2 / Conditional R^2	0.034 / 0.691			0.027 / 0.691		

eTable 15. Multivariable regression analysis of the relationship between snoring and Child Behavior Checklist Aggressive behavior score. Linear mixed-effects regression model contained the following fixed effects: age in months, biological sex at birth (comparison = female), race as self-selected by the primary caregiver (comparison = White), income in 10 classes as described in the text as an ordered factor, and time (comparison = baseline). Results are stratified by children grouped by whether they were obese or not, as defined by a body mass index (BMI) percentile ≥ 95. Random effects included the participant ID nested within the recruitment site ID. The coefficients of each predictor, their 95% confidence intervals, and the associated P values are provided. Fit is described by adjusted R^2, of which the marginal R^2 considers only the variance of the fixed effects, while the conditional R^2 considers both the fixed and random effects. The intraclass correlation coefficient (ICC) computes the proportion of the variance explained by the grouping structure in the population. τ_{00} indicates how much groups or subjects differ from each other, while the residual variance σ^2 indicates the within-subject variance. The subscripted N represents the number of children (id) and the number of sites (site).

© 2024 Isaiah A et al. *JAMA Network Open.*

Fixed effects						
	Non-obese			Obese		
Predictors	Estimates	CI	Estimates	CI	Estimates	CI
(Intercept)	47.86 ***	45.34 to 50.38	**<0.001**	46.91 ***	41.59 to 52.24	**<0.001**
Snoring	1.88 ***	1.50 to 2.27	**<0.001**	2.74 ***	2.13 to 3.35	**<0.001**
Age	0.02	-0.00 to 0.04	0.078	0.04	-0.01 to 0.09	0.094
Male	0.96 ***	0.59 to 1.32	**<0.001**	0.75	-0.03 to 1.53	0.060
Race (Black)	-3.59 ***	-4.21 to -2.97	**<0.001**	-4.72 ***	-5.73 to -3.72	**<0.001**
Race (Other)	-2.07 ***	-2.80 to -1.35	**<0.001**	-2.29 ***	-3.63 to -0.95	**0.001**
Income	-2.82 ***	-3.74 to -1.90	**<0.001**	-1.90 *	-3.48 to -0.33	**0.018**
Time	-0.48 **	-0.77 to -0.19	**0.001**	-0.53	-1.16 to 0.09	0.092
Visit type (Remote)	-1.68	-4.82 to 1.46	0.295	-0.80	-11.21 to 9.62	0.881
Visit type (Hybrid)	0.15	-0.24 to 0.53	0.457	-0.25	-1.11 to 0.60	0.560
**Random Effects**						
σ^2	38.34			43.44		
$\tau_{00\ id:site}$	65.94			74.47		
$\tau_{00\ site}$	1.03			1.12		
ICC	0.64			0.64		
N_{id}	9278			2529		
N_{site}	22			22		
Observations	26665			5800		
Marginal R^2 / Conditional R^2	0.026 / 0.645			0.051 / 0.654		

eTable 16. Multivariable regression analysis of the relationship between snoring and Child Behavior Checklist Internalizing problems score. Linear mixed-effects regression model contained the following fixed effects: age in months, biological sex at birth (comparison = female), race as self-selected by the primary caregiver (comparison = White), income in 10 classes as described in the text as an ordered factor, and time (comparison = baseline). Results are stratified by children grouped by whether they were obese or not, as defined by a body mass index (BMI) percentile ≥ 95. Random effects included the participant ID nested within the recruitment site ID. The coefficients of each predictor, their 95% confidence intervals, and the associated P values are provided. Fit is described by adjusted R^2, of which the marginal R^2 considers only the variance of the fixed effects, while the conditional R^2 considers both the fixed and random effects. The intraclass correlation coefficient (ICC) computes the proportion of the variance explained by the grouping structure in the population. τ_{00} indicates how much groups or subjects differ from each other, while the residual variance σ^2 indicates the within-subject variance. The subscripted N represents the number of children (id) and the number of sites (site).

© 2024 Isaiah A et al. *JAMA Network Open.*

Fixed effects						
	Non-obese			Obese		
Predictors	Estimates	CI	Estimates	CI	Estimates	CI
(Intercept)	48.77 ***	46.37 to 51.16	**<0.001**	47.73 ***	42.62 to 52.85	**<0.001**
Snoring	1.82 ***	1.47 to 2.16	**<0.001**	2.23 ***	1.69 to 2.77	**<0.001**
Age	-0.01	-0.03 to 0.01	0.287	0.01	-0.04 to 0.06	0.733
Male	1.36 ***	1.01 to 1.72	**<0.001**	0.43	-0.33 to 1.19	0.267
Race (Black)	-1.28 ***	-1.88 to -0.68	**<0.001**	-2.28 ***	-3.26 to -1.30	**<0.001**
Race (Other)	-1.86 ***	-2.56 to -1.16	**<0.001**	-2.14 **	-3.45 to -0.84	**0.001**
Income	-6.02 ***	-6.90 to -5.14	**<0.001**	-4.21 ***	-5.75 to -2.68	**<0.001**
Time	-0.27	-0.54 to 0.01	0.055	-0.53	-1.12 to 0.06	0.077
Visit type (Remote)	0.18	-2.57 to 2.92	0.900	-3.85	-12.83 to 5.13	0.401
Visit type (Hybrid)	0.24	-0.09 to 0.57	0.158	-0.33	-1.07 to 0.42	0.389
**Random Effects**						
σ^2	28.77			31.93		
$\tau_{00\ id:site}$	64.51			74.77		
$\tau_{00\ site}$	0.83			1.46		
ICC	0.69			0.70		
N_{id}	9278			2529		
N_{site}	22			22		
Observations	26665			5800		
Marginal R^2 / Conditional R^2	0.038 / 0.706			0.033 / 0.715		

eTable 17. Multivariable regression analysis of the relationship between snoring and Child Behavior Checklist Externalizing problems score. Linear mixed-effects regression model contained the following fixed effects: age in months, biological sex at birth (comparison = female), race as self-selected by the primary caregiver (comparison = White), income in 10 classes as described in the text as an ordered factor, and time (comparison = baseline). Results are stratified by children grouped by whether they were obese or not, as defined by a body mass index (BMI) percentile ≥ 95. Random effects included the participant ID nested within the recruitment site ID. The coefficients of each predictor, their 95% confidence intervals, and the associated P values are provided. Fit is described by adjusted R^2, of which the marginal R^2 considers only the variance of the fixed effects, while the conditional R^2 considers both the fixed and random effects. The intraclass correlation coefficient (ICC) computes the proportion of the variance explained by the grouping structure in the population. τ_{00} indicates how much groups or subjects differ from each other, while the residual variance σ^2 indicates the within-subject variance. The subscripted N represents the number of children (id) and the number of sites (site).

© 2024 Isaiah A et al. *JAMA Network Open.*

Fixed effects						
	Non-obese			Obese		
Predictors	Estimates	CI	Estimates	CI	Estimates	CI
(Intercept)	47.99 ***	45.29 to 50.69	**<0.001**	47.12 ***	41.43 to 52.81	**<0.001**
Snoring	2.40 ***	2.03 to 2.77	**<0.001**	3.18 ***	2.59 to 3.77	**<0.001**
Age	-0.00	-0.03 to 0.02	0.818	0.02	-0.03 to 0.07	0.396
Male	1.46 ***	1.06 to 1.86	**<0.001**	0.65	-0.20 to 1.49	0.133
Race (Black)	-2.71 ***	-3.39 to -2.04	**<0.001**	-4.21 ***	-5.31 to -3.11	**<0.001**
Race (Other)	-2.60 ***	-3.39 to -1.81	**<0.001**	-2.82 ***	-4.28 to -1.36	**<0.001**
Income	-4.87 ***	-5.87 to -3.87	**<0.001**	-2.92 ***	-4.63 to -1.21	**0.001**
Time	-0.32 *	-0.62 to -0.01	**0.042**	-0.52	-1.17 to 0.13	0.119
Visit type (Remote)	-1.41	-4.34 to 1.53	0.347	-1.47	-11.26 to 8.33	0.769
Visit type (Hybrid)	0.10	-0.25 to 0.46	0.563	-0.26	-1.07 to 0.55	0.534
**Random Effects**						
σ^2	32.63			37.92		
$\tau_{00\ id:site}$	83.55			93.77		
$\tau_{00\ site}$	1.60			2.12		
ICC	0.72			0.72		
N_{id}	9278			2529		
N_{site}	22			22		
Observations	26665			5800		
Marginal R^2 / Conditional R^2	0.034 / 0.733			0.046 / 0.730		

eTable 18. Multivariable regression analysis of the relationship between snoring and Child Behavior Checklist Total problems score. Linear mixed-effects regression model contained the following fixed effects: age in months, biological sex at birth (comparison = female), race as self-selected by the primary caregiver (comparison = White), income in 10 classes as described in the text as an ordered factor, and time (comparison = baseline). Results are stratified by children grouped by whether they were obese or not, as defined by a body mass index (BMI) percentile ≥ 95. Random effects included the participant ID nested within the recruitment site ID. The coefficients of each predictor, their 95% confidence intervals, and the associated P values are provided. Fit is described by adjusted R^2, of which the marginal R^2 considers only the variance of the fixed effects, while the conditional R^2 considers both the fixed and random effects. The intraclass correlation coefficient (ICC) computes the proportion of the variance explained by the grouping structure in the population. τ_{00} indicates how much groups or subjects differ from each other, while the residual variance σ^2 indicates the within-subject variance. The subscripted N represents the number of children (id) and the number of sites (site).

© 2024 Isaiah A et al. *JAMA Network Open.*
