# Supplementary material for: Cognitive and Behavioral Outcomes of Snoring Among Adolescents
Source: JAMA Netw Open. 2024 Nov 8;7(11):e2444057. doi: 10.1001/jamanetworkopen.2024.44057 (PMC11549662; doi:10.1001/jamanetworkopen.2024.44057)
Supplement: Supplement 2. — Data Sharing Statement [file jamanetwopen-e2444057-s002.pdf]

Data Sharing Statement

Isaiah. Cognitive and Behavioral Outcomes of Snoring Among Adolescents. *JAMA Netw Open*. Published November 08, 2024. doi:10.1001/jamanetworkopen.2024.44057

Data
Data available: No

Additional Information
Explanation for why data not available: Data can be obtained directly from the NIMH following a data use agreement. Secondary sharing is not allowed. All statistical code and de-identified data frames can be shared upon request.
